# Supplementary material for: CNETML: maximum likelihood inference of phylogeny from copy number profiles of multiple samples
Source: Genome Biol. 2023 Jun 20;24:144. doi: 10.1186/s13059-023-02983-0 (PMC10283241; doi:10.1186/s13059-023-02983-0)
Supplement: Supplementary file 1 — Additional file 1: Supplementary file. Supplementary Tables and Figures. [file 13059_2023_2983_MOESM1_ESM.pdf]

Supplementary document for CNETML: maximum likelihood inference of phylogeny from copy number profiles of multiple samples

Table S1: The rate matrix Q when the maximum total copy number $c_{max} = 4$.

	**0**	**1**	**2**	**3**	**4**	**5**	**6**	**7**	**8**	**9**	**10**	**11**	**12**	**13**	**14**
	**0/0**	**0/1**	**1/0**	**0/2**	**1/1**	**2/0**	**0/3**	**1/2**	**2/1**	**3/0**	**0/4**	**1/3**	**2/2**	**3/1**	**4/0**
**0/0**	0	0	0	0	0	0	0	0	0	0	0	0	0	0	0
**0/1**	e	$-(u+e)$	0	u	0	0	0	0	0	0	0	0	0	0	0
**1/0**	e	0	$-(u+e)$	0	0	u	0	0	0	0	0	0	0	0	0
**0/2**	0	e	0	$-(u+e)$	0	0	u	0	0	0	0	0	0	0	0
**1/1**	0	e	e	0	$-2(u+e)$	0	0	u	u	0	0	0	0	0	0
**2/0**	0	0	e	0	0	$-(u+e)$	0	0	0	u	0	0	0	0	0
**0/3**	0	0	0	e	0	0	$-(u+e)$	0	0	0	u	0	0	0	0
**1/2**	0	0	0	e	e	0	0	$-2(u+e)$	0	0	0	u	u	0	0
**2/1**	0	0	0	0	e	e	0	0	$-2(u+e)$	0	0	0	u	u	0
**3/0**	0	0	0	0	0	e	0	0	0	$-(u+e)$	0	0	0	0	u
**0/4**	0	0	0	0	0	0	e	0	0	0	$-e$	0	0	0	0
**1/3**	0	0	0	0	0	0	e	e	0	0	0	$-2e$	0	0	0
**2/2**	0	0	0	0	0	0	0	e	e	0	0	0	$-2e$	0	0
**3/1**	0	0	0	0	0	0	0	0	e	e	0	0	0	$-2e$	0
**4/0**	0	0	0	0	0	0	0	0	0	e	0	0	0	0	$-e$

Table S2: Parameters used for tree generation in CNETS.

effective population size	N_e	90000
generation time in year (365 days)	t	0.002739726
exponential growth rate	β	1.563e-3

Table S3: The summary statistics on the numbers of (unique) variant sites in the data simulated by CNETS for Fig. 3 and the numbers of reconstructed trees with correct topologies in Fig. 3.

**number of sites**	**copy number type**	**mutation rate (per haplotype per site per year)**	**range of the number of variant sites**	**range of the number of unique variant sites**	**number of reconstructed trees with correct topologies**
100	total	0.001	5, 25	5, 14	42
		0.01	62, 85	28, 55	58
	haplotype-specific	0.001	5, 25	5, 17	41
		0.01	62, 88	37, 75	76
1000	total	0.001	90, 180	16, 32	82
		0.01	614, 830	116, 235	83
	haplotype-specific	0.001	90, 180	28, 49	85
		0.01	620, 841	196, 437	90
10000	total	0.001	873, 1706	41, 86	97
		0.01	6520, 8223	345, 642	100
	haplotype-specific	0.001	876, 1710	69, 165	98
		0.01	6612, 8329	947, 2039	99

Table S4: The number of cases where distances of trees reconstructed by heuristic search to simulated trees were no larger than those of trees reconstructed by exhaustive search on data simulated with different mutation rates and number of samples.

**mutation rate (per haplotype per site per year)**	**number of samples**	**number of cases where heuristic tree search generated smaller branch score distances**	**number of cases where heuristic tree search generated the same (smaller) normalized RF distances**
0.001	5	50	100
	6	52	99
	7	37	68 (1)
0.01	5	53	100
	6	42	91 (3)
	7	41	73 (3)

Table S5: The data simulated by CNETS under different temporal signal strengths, grouped by the mean pairwise absolute difference of tip relative times.

**group**	**time range (year)**	**mutation rate (per haplotype per site per year)**	**number of sites**	**number of samples**
small	[0.4, 3)	0.001	1000	103
			10000	106
		0.01	1000	106
			10000	103
intermediate	[3, 7)	0.001	1000	108
			10000	99
		0.01	1000	104
			10000	108
high	[7, 14]	0.001	1000	89
			10000	95
		0.01	1000	90
			10000	89

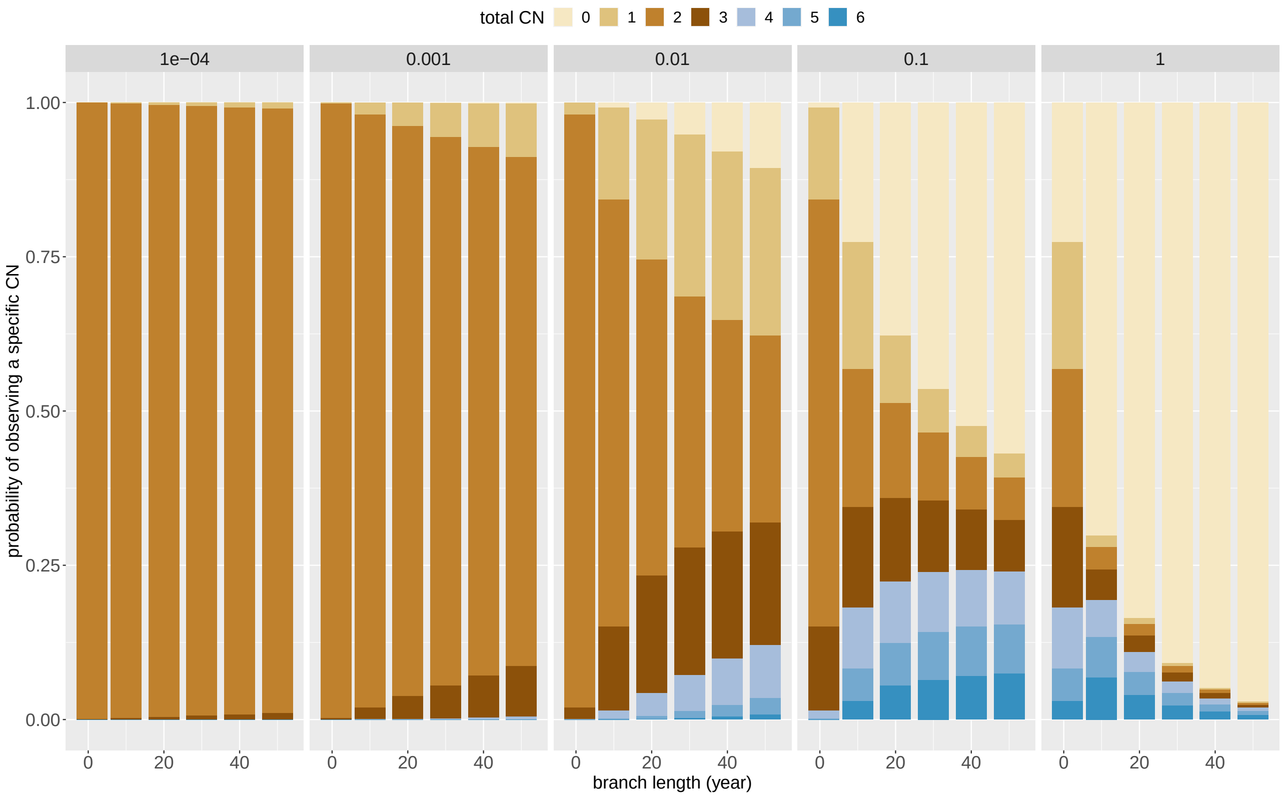

Fig. S1: The distribution of total copy number (CN) at the end of one branch under the Markov model. The plots are grouped by mutation rates. In each group, the x-axis shows branch length at size 1, 10, 20, ..., 50. The y-axis shows the probability of changing from normal total copy number (2) to each possible total copy number. We computed the final states of the Markov chain for one branch of varying lengths starting at normal state, copy number (1,1). When the mutation rate is very low (1e-4 per haplotype per site per year), there are only a few mutations and most sites stay normal. When the mutation rate is high (1 per haplotype per site per year), more sites reach absorbing states (copy number 0).

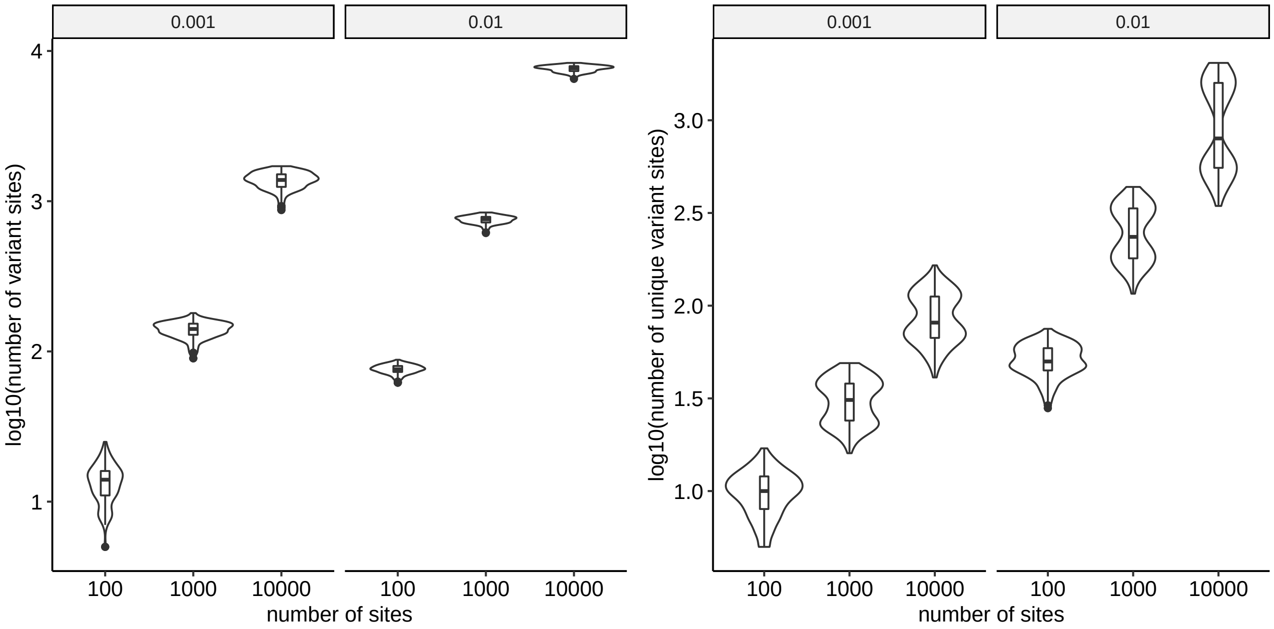

Fig. S2: The distribution of the numbers of (unique) variant sites in the data simulated by CNETS for Fig. 3. All the simulated samples are at the same time point. The plots are grouped by mutation rates. There are 100 datasets for each parameter setting. The box plots show the median (centre), 1st (lower hinge), and 3rd (upper hinge) quartiles of the data; the whiskers extend to 1.5× of the interquartile range (distance between the 1st and 3rd quartiles); data beyond the interquartile range are plotted individually.

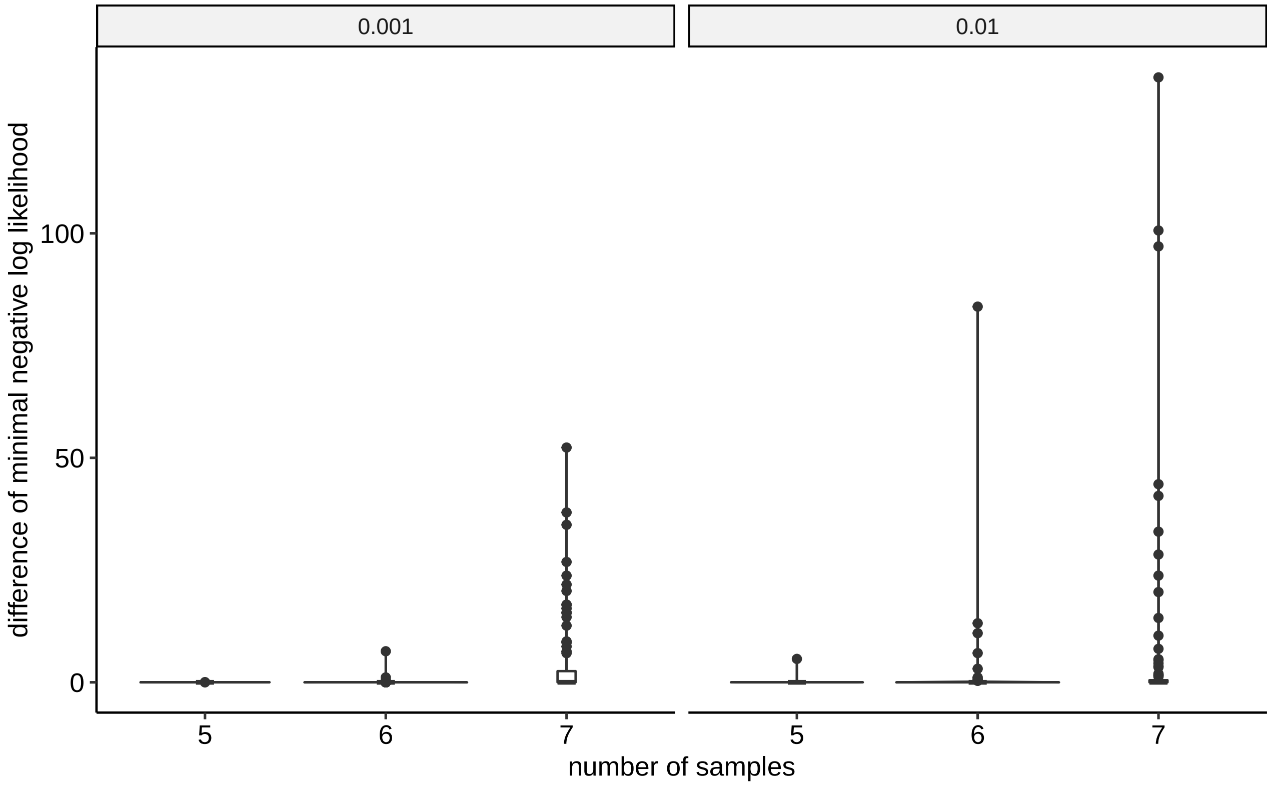

Fig. S3: The difference of minimal negative log likelihood values of CNETML when using heuristic and exhaustive tree search on data simulated with different mutation rates and a small number of samples. All the simulated samples are at the same time point. The data with five samples are the same as those in Fig. 3. Box plots have the same interpretations as those in Fig. S2.

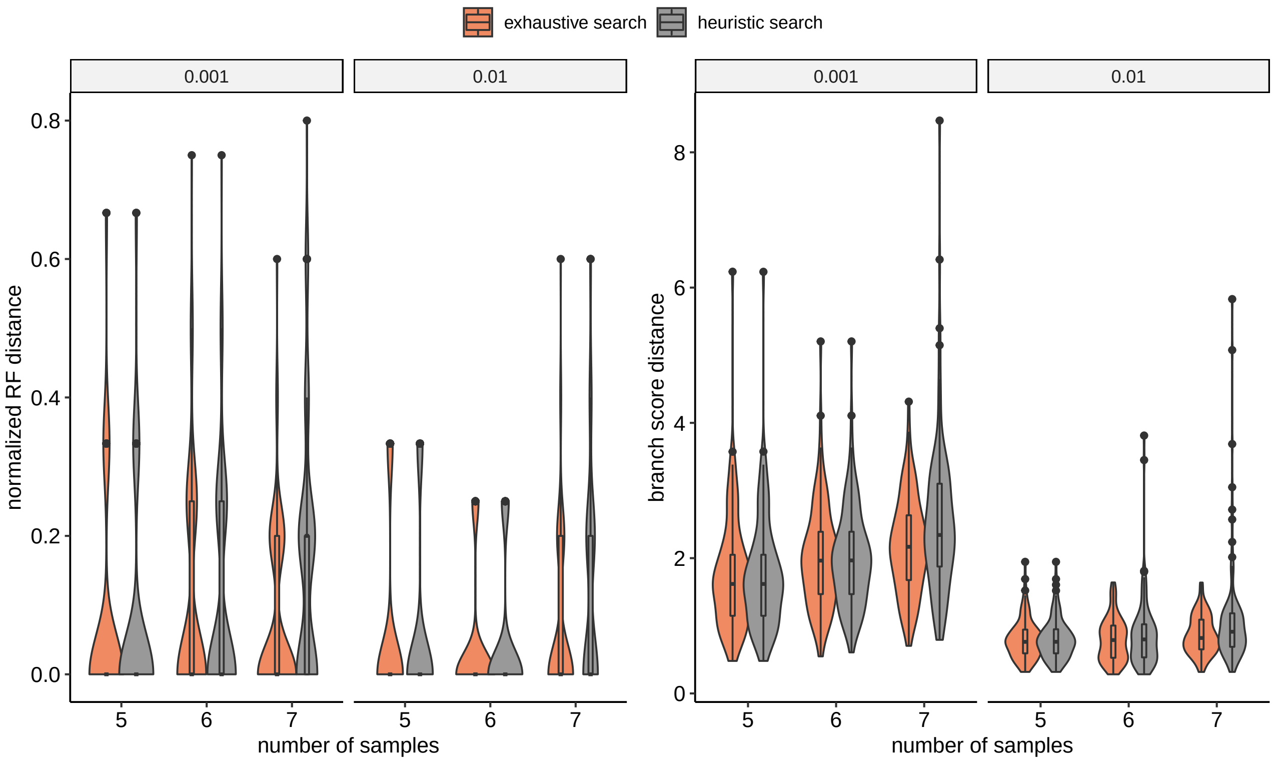

Fig. S4: The performance of CNETML when using exhaustive and heuristic tree search on data simulated with different mutation rates and a small number of samples. The same data as those in Fig. S3. Box plots have the same interpretations as those in Fig. S2.

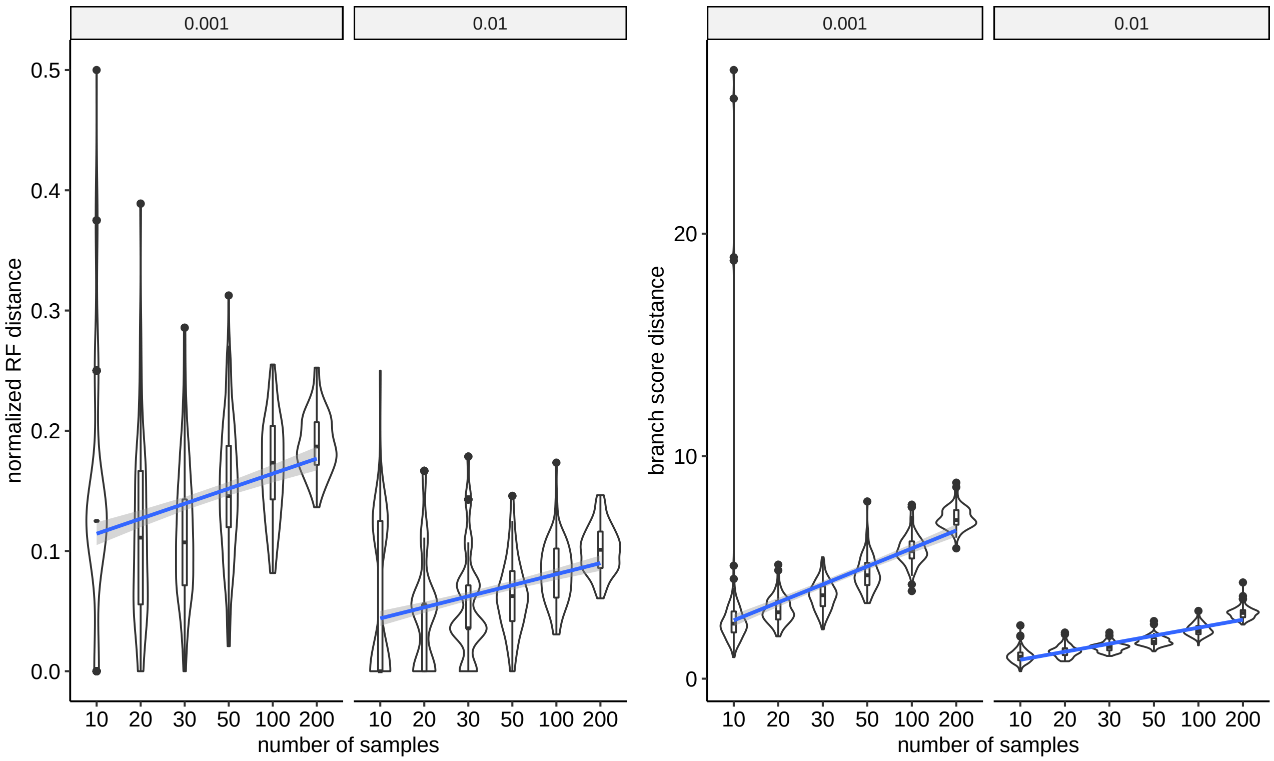

Fig. S5: The performance of CNETML (heuristic tree search) on data simulated with different mutation rates and number of samples. All the simulated samples are at the same time point. The plots are grouped by mutation rates. There are 100 datasets for each parameter setting except that there are 85 (87) datasets when mutation rate is 0.001 (0.01) per haplotype per site per year and the number of samples is 200 (due to a computation time limit of 120 hours). Box plots have the same interpretations as those in Fig. S2.

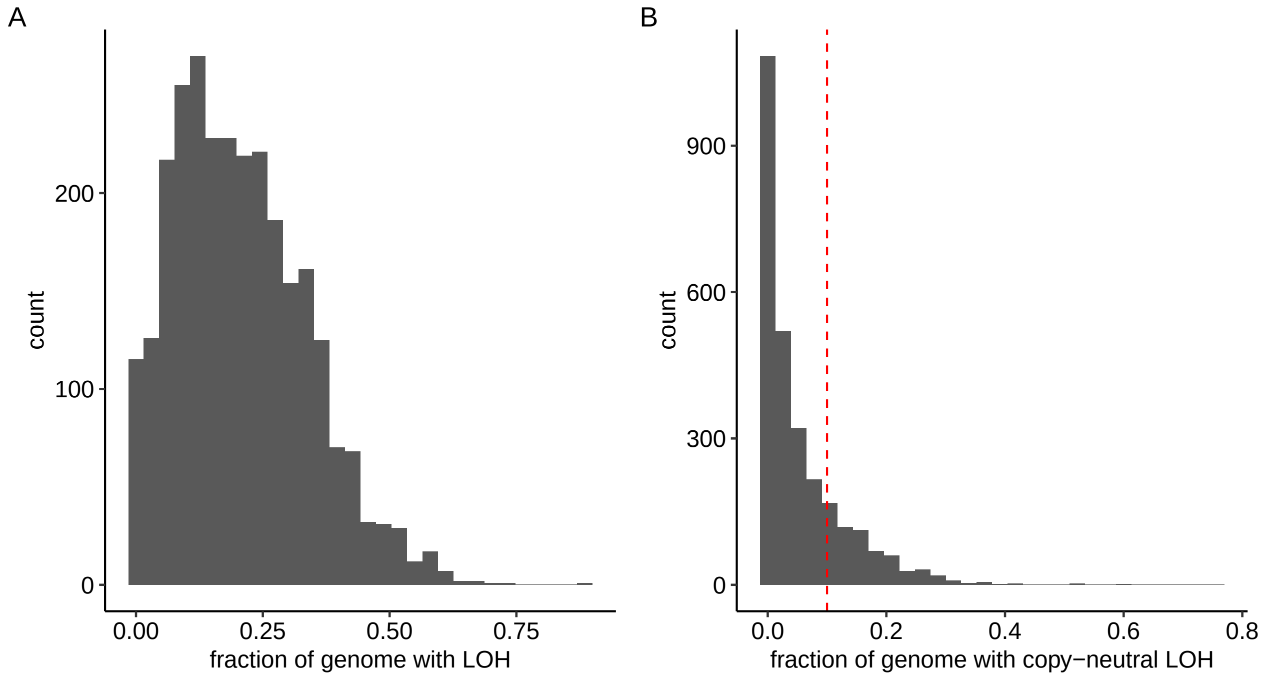

Fig. S6: The distribution of the fractions of genome with loss of heterozygosity (LOH) on 2,778 samples from PCAWG dataset. **A**: The distribution of the fractions of genome with LOH. **B**: The distribution of the fractions of genome with copy-neutral LOH (red dashed line: 0.1).

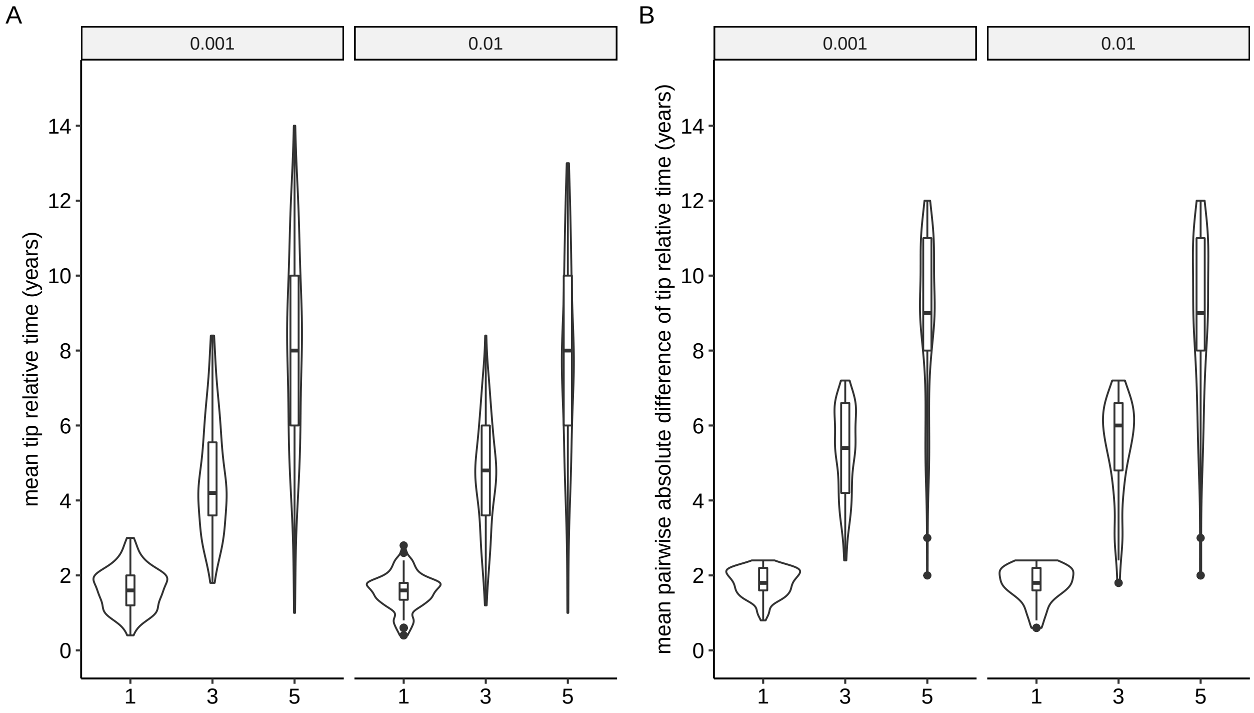

Fig. S7: The range of simulated sampling times under different temporal signal strengths and mutation rates. **A**: The average of the relative times at the tips (assuming the first sample is at time 0) in the simulated trees. **B**: The average of pairwise absolute difference of the relative times at the tips in the simulated trees. The x-axis shows the value of *dt* which controls temporal signal strength, with larger value indicating larger time differences among samples. The plots are grouped by mutation rates. There are five samples in each simulated tree and 100 datasets for each parameter setting. Box plots have the same interpretations as those in Fig. S2.

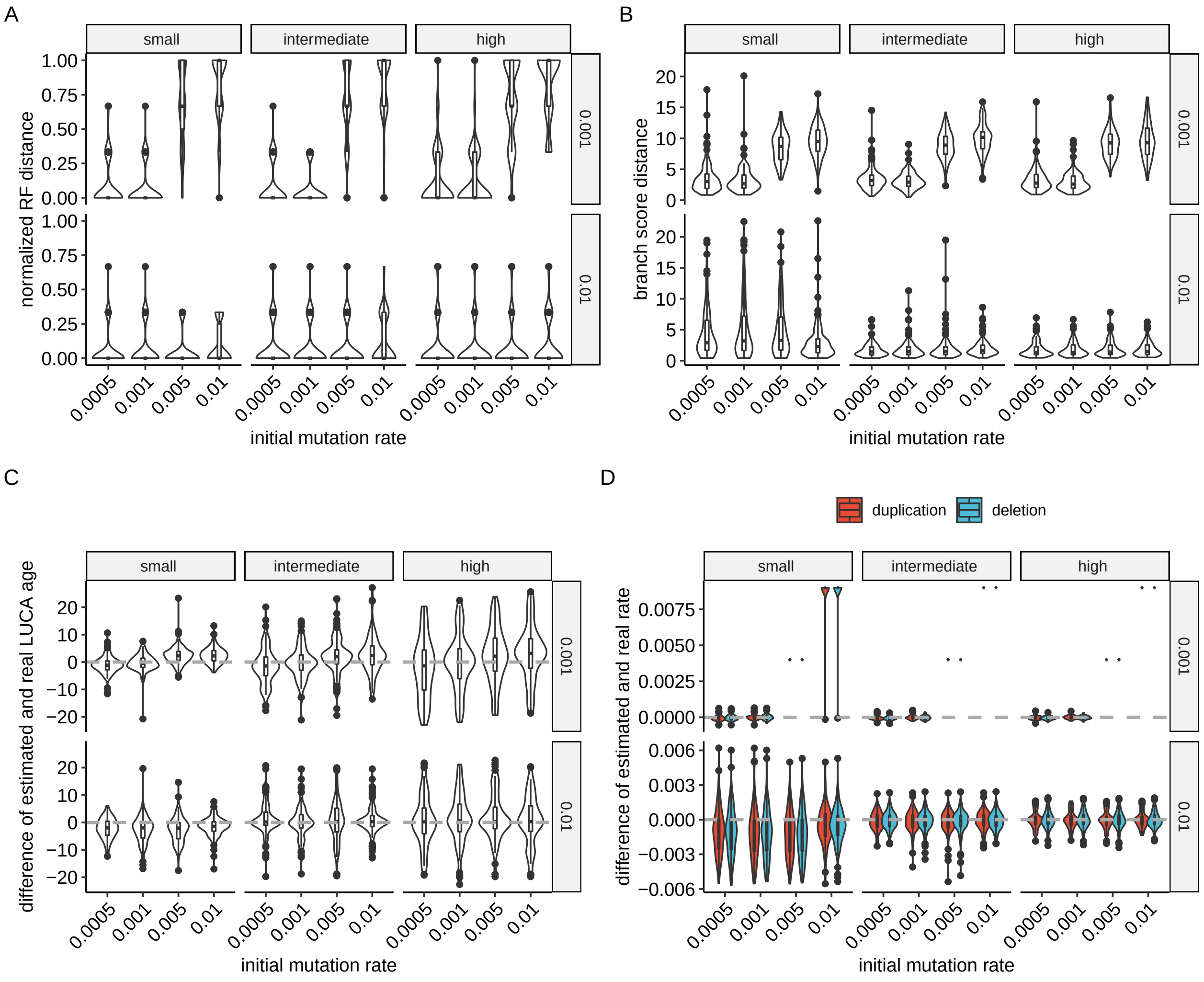

Fig. S8: The sensitivity of CNETML to initial mutation rates when jointly estimating the tree topology, node ages, and mutation rates on data simulated with different temporal signal strengths and mutation rates. **A-C**: The accuracy of tree inference under different initial mutation rates. **D**: The accuracy of mutation rate estimation under different initial mutation rates. There are five samples in each simulated tree and 100 datasets for each parameter setting. Box plots have the same interpretations as those in Fig. S2.

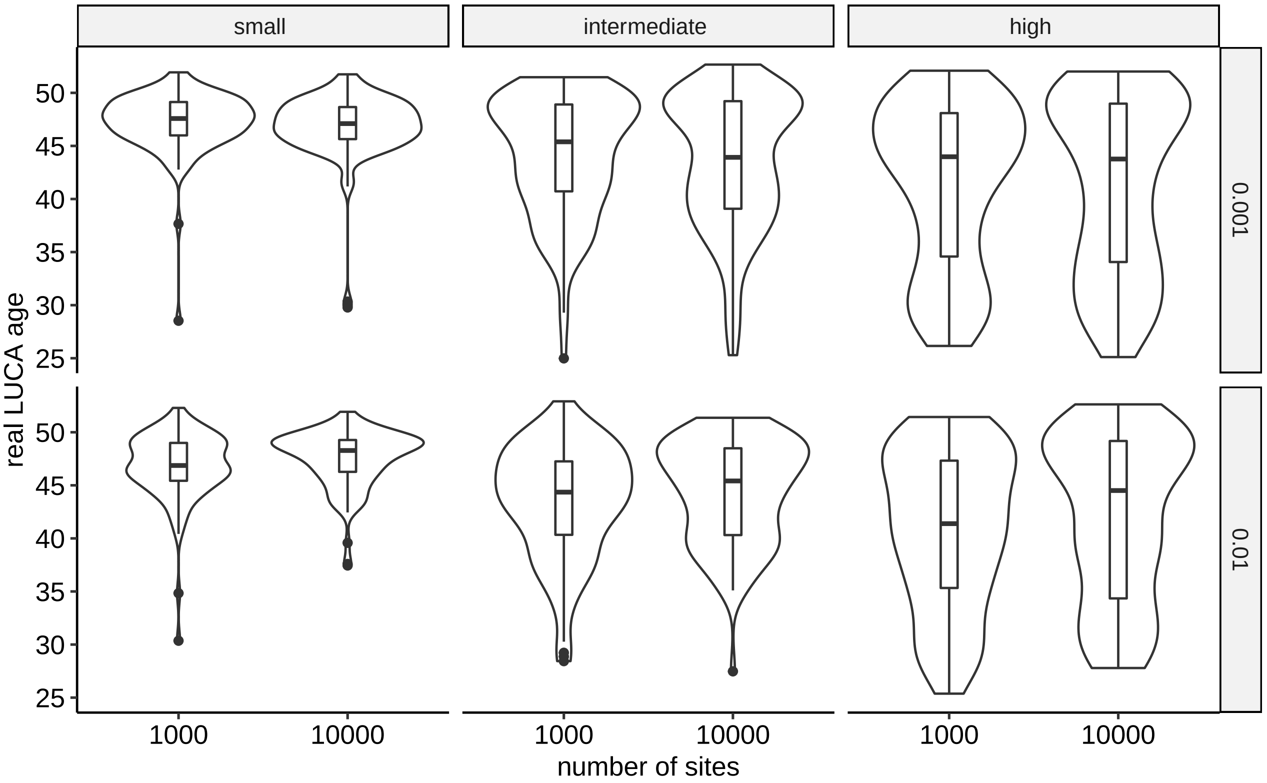

Fig. S9: The simulated real LUCA ages under different temporal signal strengths and mutation rates. The plots are grouped by mutation rates and sampling time differences. There are five samples in each simulated tree and 100 datasets for each parameter setting. Box plots have the same interpretations as those in Fig. S2.

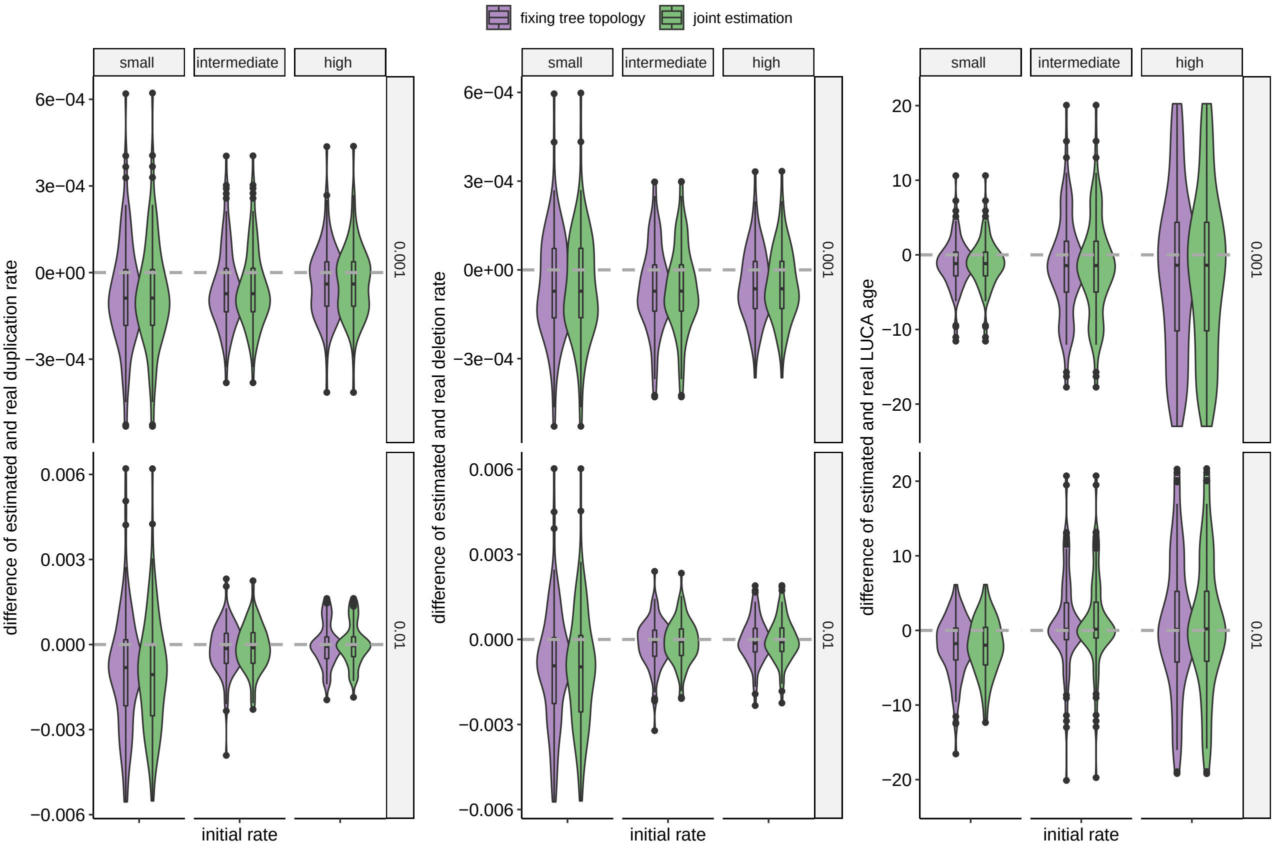

Fig. S10: The differences of parameter estimations with the tree topology fixed or not. The plots are grouped by mutation rates and sampling time differences. There are five samples in each simulated tree and 100 datasets for each parameter setting, which are the same as the data with 1,000 sites in Fig. 4. Box plots have the same interpretations as those in Fig. S2.

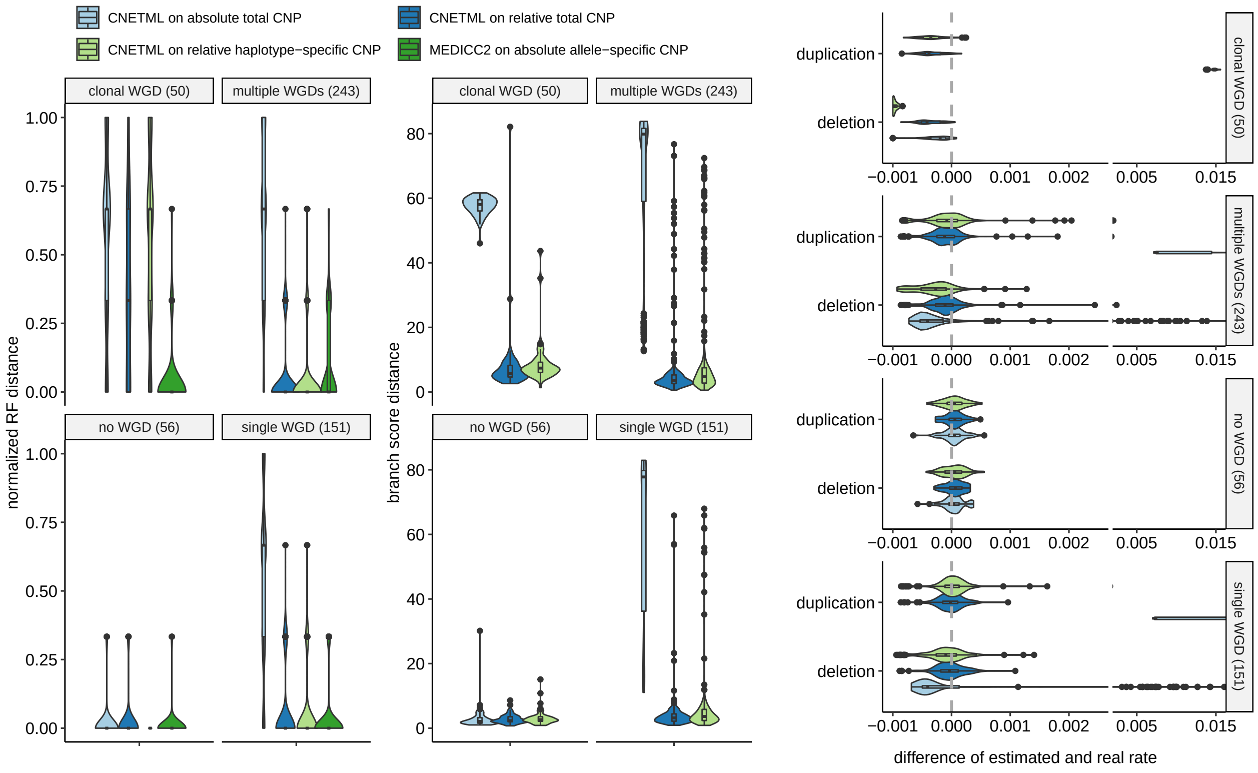

Fig. S11: The performance of CNETML on relative copy number data obtained by using baseline based on the number of WGDs and random rounding. There are 500 simulated datasets in total, which are divided into four groups by the types of WGDs. The number of datasets in each group is shown in brackets. MEDICC2 was excluded when comparing branch score distance because the branch length in a tree built by it has a different meaning (the number of events between CNPs of two nodes based on CNT model) and it is hard to compare fairly. 91 outlier data points with values larger than the maximum of x-axis on datasets with subclonal WGDs are excluded in the plot of mutation rates for better visualization of the majority data. Box plots have the same interpretations as those in Fig. S2.

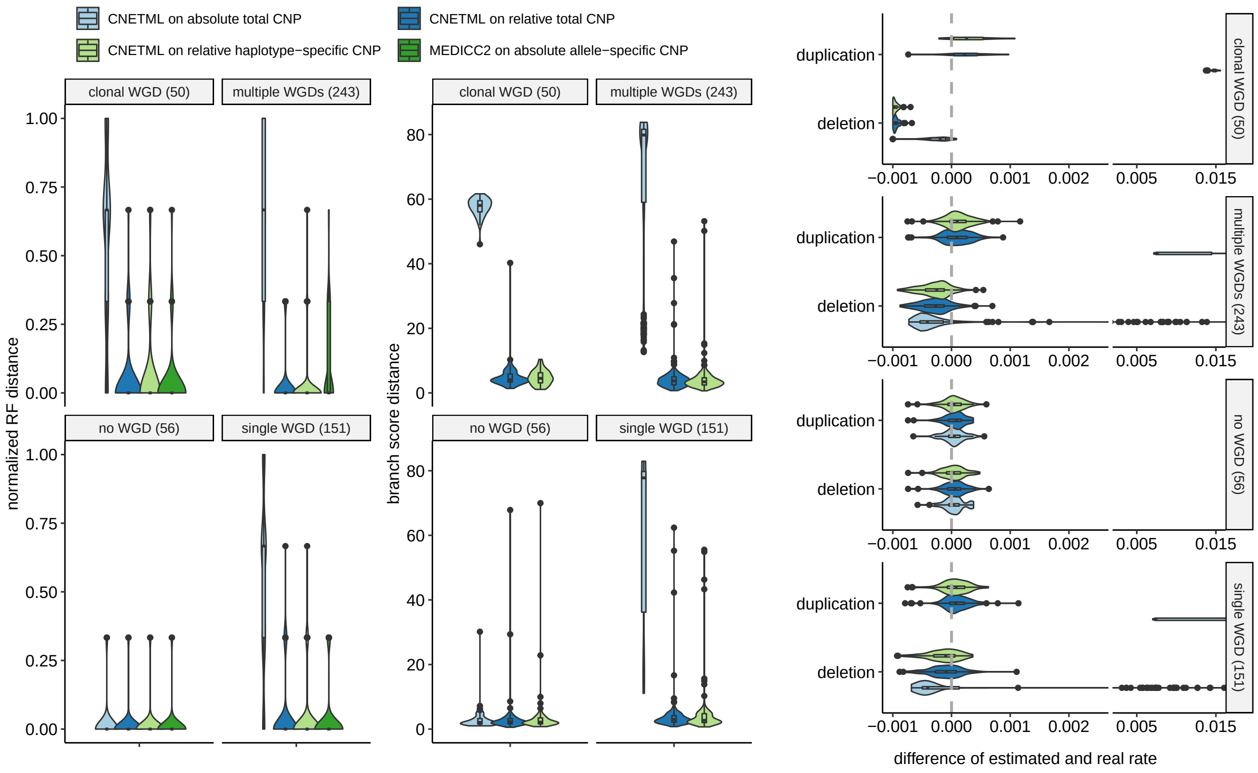

Fig. S12: The performance of CNETML on relative copy number data obtained by using baseline based on the number of WGDs and direct rounding. The data are the same as those in Fig. S11 except the relative copy numbers. Box plots have the same interpretations as those in Fig. S2.

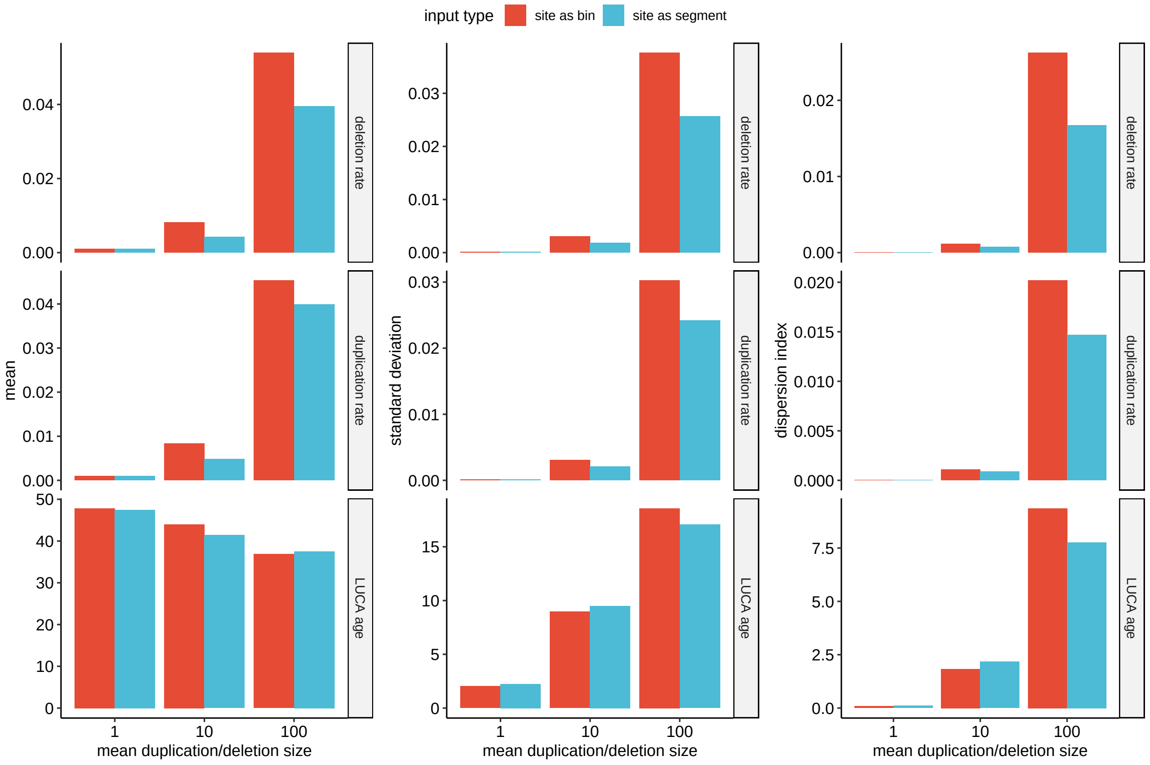

Fig. S13: The mean, standard deviation, and dispersion index (variance to mean ratio) of the estimated mutation rates and LUCA age when building trees with different types of sites on data simulated with mean duplication/deletion of different sizes. There are five samples in each simulated tree and 100 datasets for each parameter setting, which are the same as the data in Fig. 6.

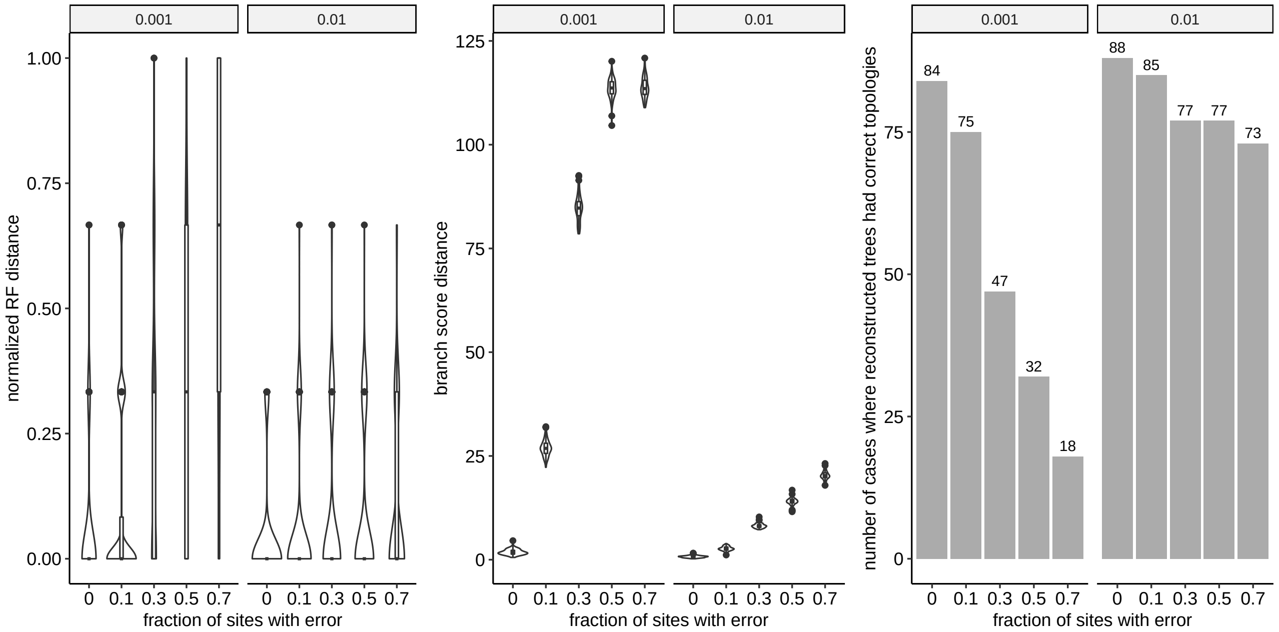

Fig. S14: The performance of CNETML at increasing error rates when the samples were at the same time point. The plots are grouped by mutation rates. There are five samples in each simulated tree and 100 datasets for each parameter setting. Box plots have the same interpretations as those in Fig. S2.

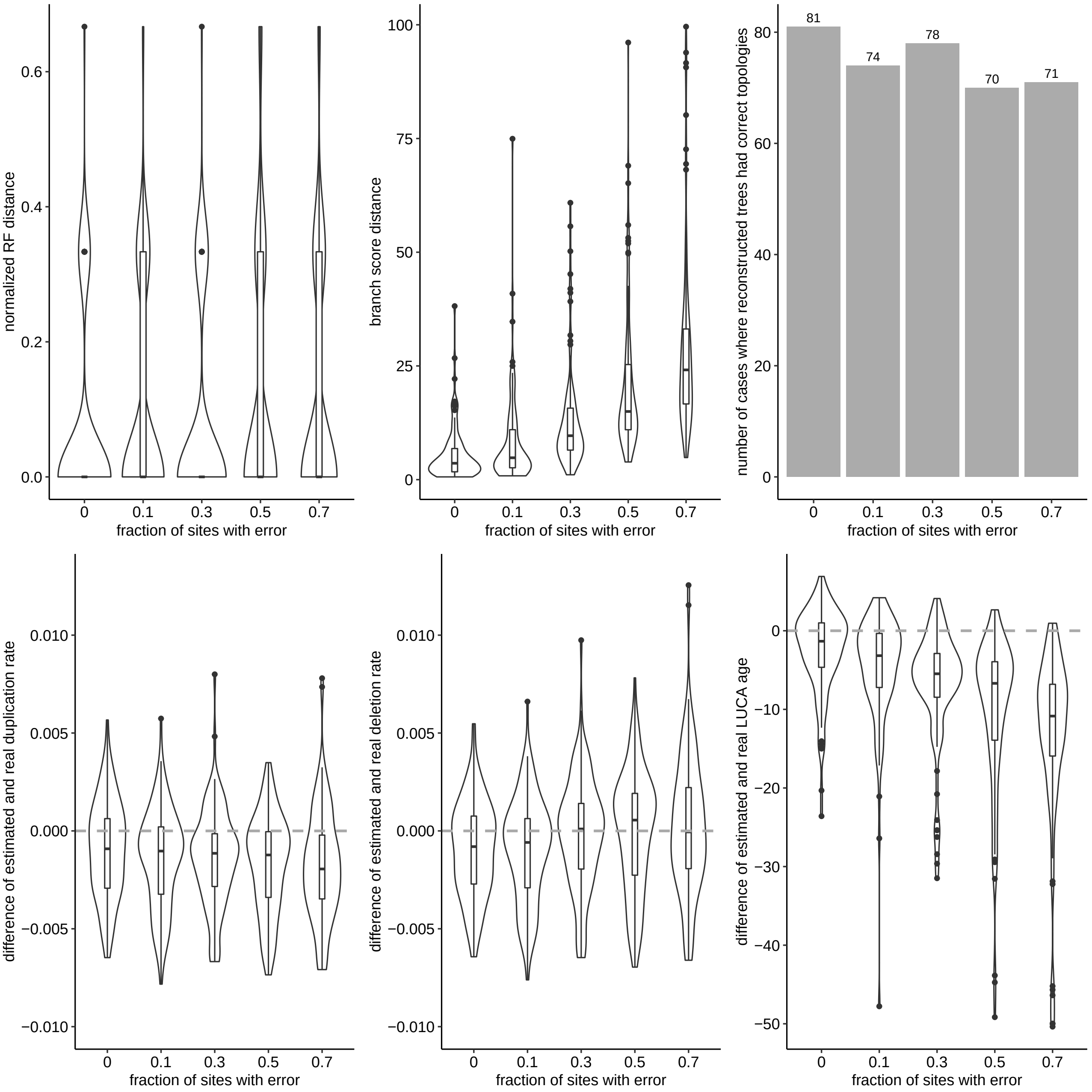

Fig. S15: The performance of CNETML at increasing error rates when the samples were at different time points. The data were simulated with $dt = 1$ year and duplication/deletion rate 0.01 per haplotype per site per year. The initial value for duplication/deletion rate estimation was set to 0.0005 (per haplotype per site per year). There are five samples in each simulated tree and 100 datasets for each parameter setting. Box plots have the same interpretations as those in Fig. S2.

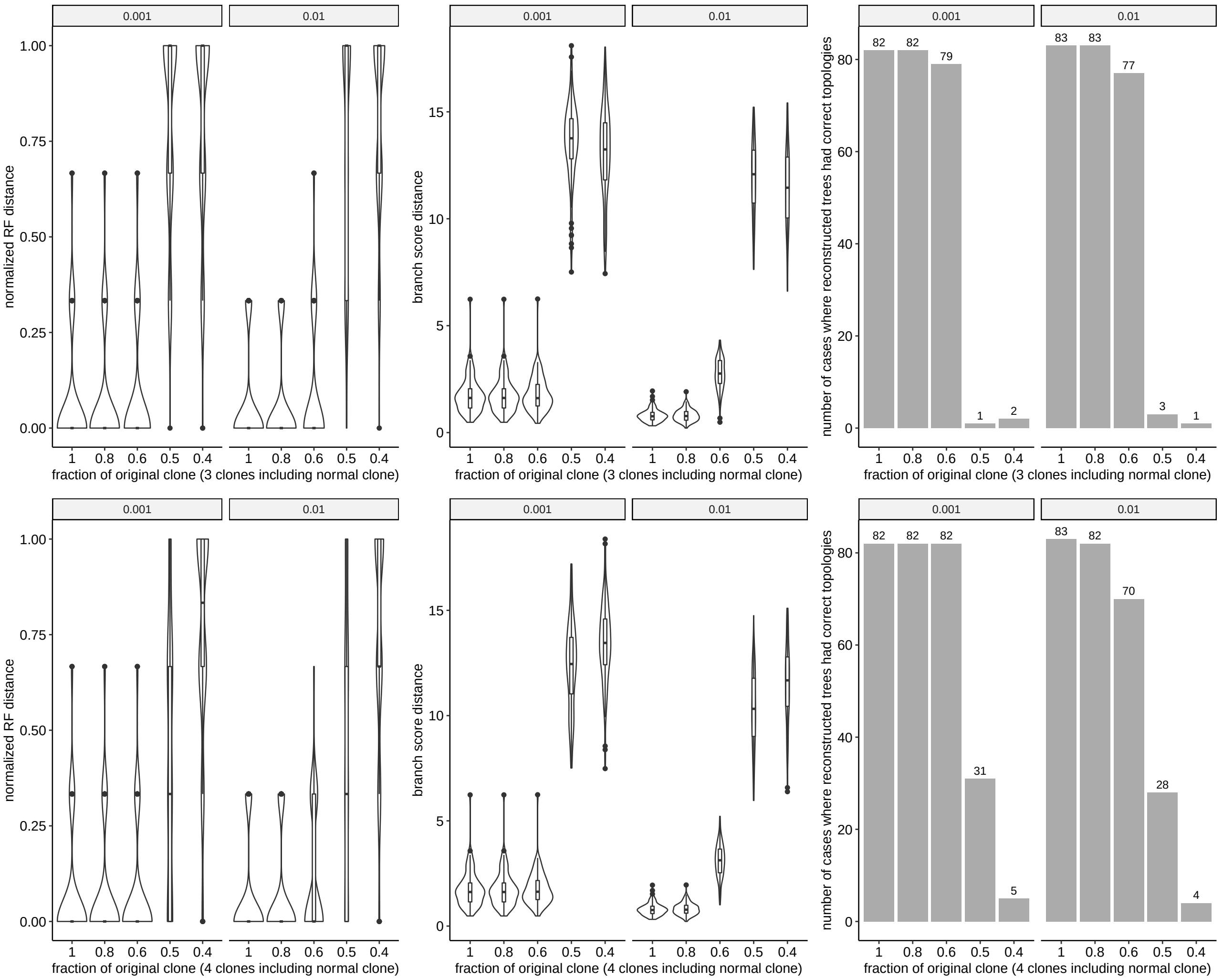

Fig. S16: The performance of CNETML under violation of sample homogeneity when the samples were at the same time point. Here, "original clone" is the clone with the same identifier as the sample. The data with no subclone (fraction of orginal clone being 1) is from the data in Fig. 3. The plots are grouped by mutation rates. There are five samples in each simulated tree and 100 datasets for each parameter setting. Box plots have the same interpretations as those in Fig. S2.

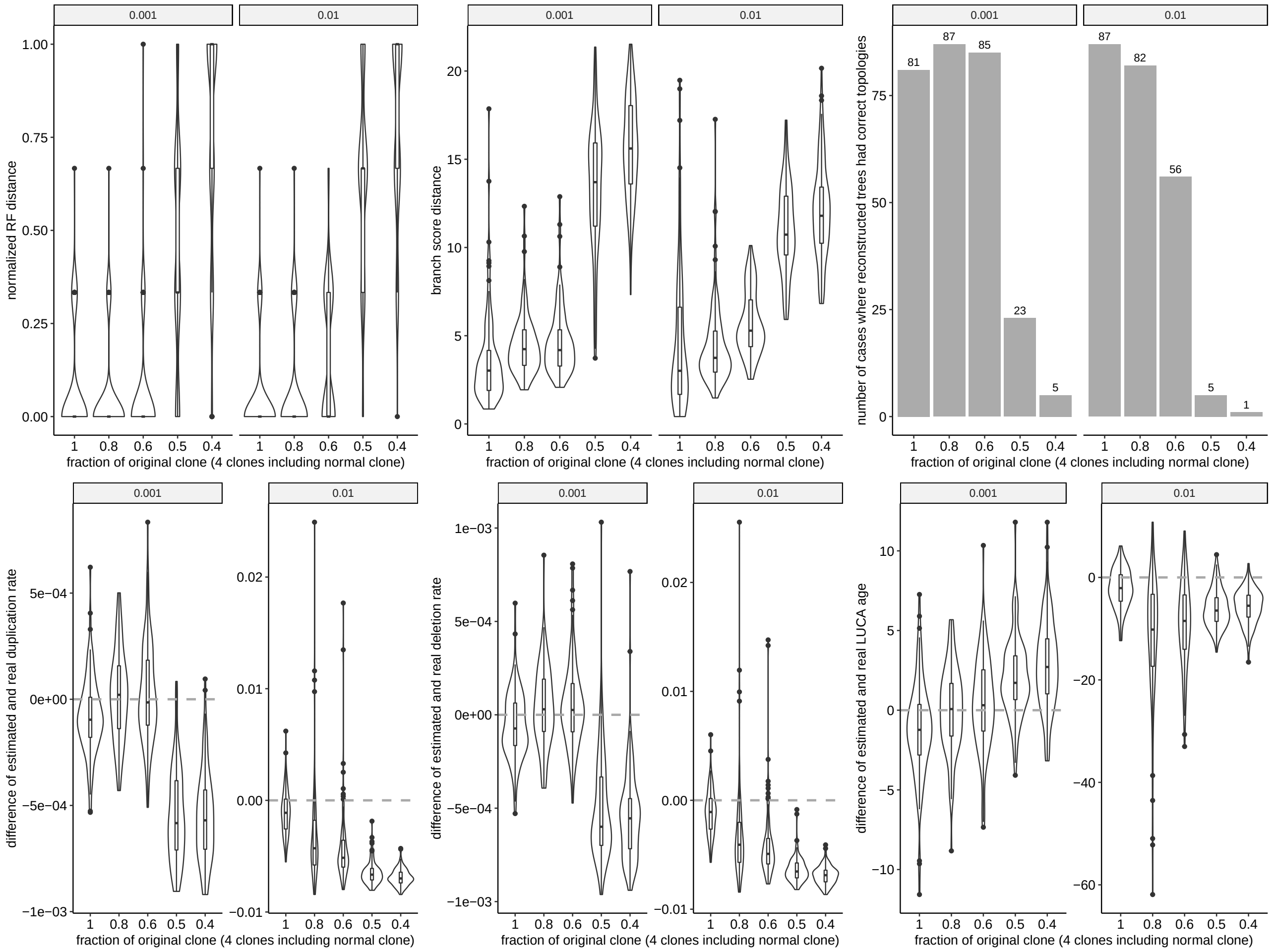

Fig. S17: The performance of CNETML under violation of sample homogeneity when the samples were at different time points. Here, "original clone" is the clone with the same identifier as the sample. The data were simulated with $dt = 1$ year. The data with no subclone (fraction of orginal clone being 1) is from the data in Fig. 4. The initial value for duplication/deletion rate estimation was set to 0.0005 per haplotype per site per year. The plots are grouped by mutation rates. There are five samples in each simulated tree and 100 datasets for each parameter setting. Box plots have the same interpretations as those in Fig. S2.

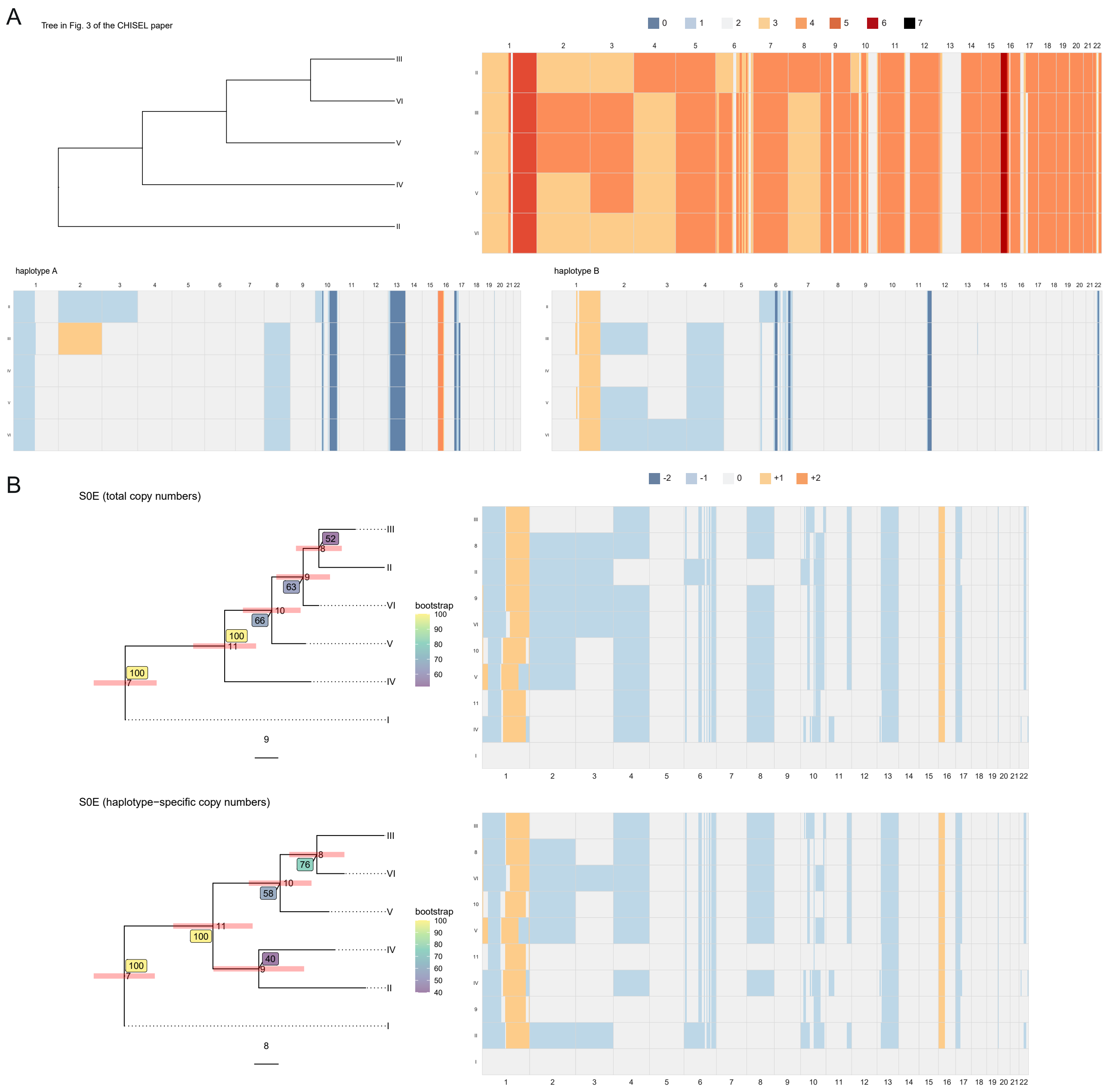

Fig. S18: The performance of CNETML on 6 clones (5 tumour clones and one diploid clone I) detected from 1,448 cells in section E of patient S0. **A**: The phylogenetic tree and heatmaps of copy number data based on Fig. 3 in [1]. **B**: The phylogenetic trees reconstructed by CNETML on relative total and haplotype-specific copy numbers, respectively. The bootstrap support values are shown in coloured rectangles with lighter colours suggesting stronger support. The coloured bars at the internal nodes show the confidence intervals of the expected number of copy number alterations. When using relative total copy numbers as input, clone II in the tree inferred by CNETML has a different position from the tree in [1] (bootstrap support 52%). When using relative haplotype-specific copy numbers of data as input, clone IV in the tree inferred by CNETML has a different position from the tree in [1] (bootstrap support 40%). In [1], the events separating clone II (chr2.A del, chr3.A del) and the other clones (chr2.B del, chr3.B del) are mainly the mirrored-subclonal CNAs on chromosome 2 and 3. The overall consistency and uncertainty in placing clone II or clone IV (relative to clone II) suggest that CNETML can reconstruct reasonable phylogenies from tumour clones detected from single cell data. The trees were reconstructed in a similar approach to that in Fig. 7.

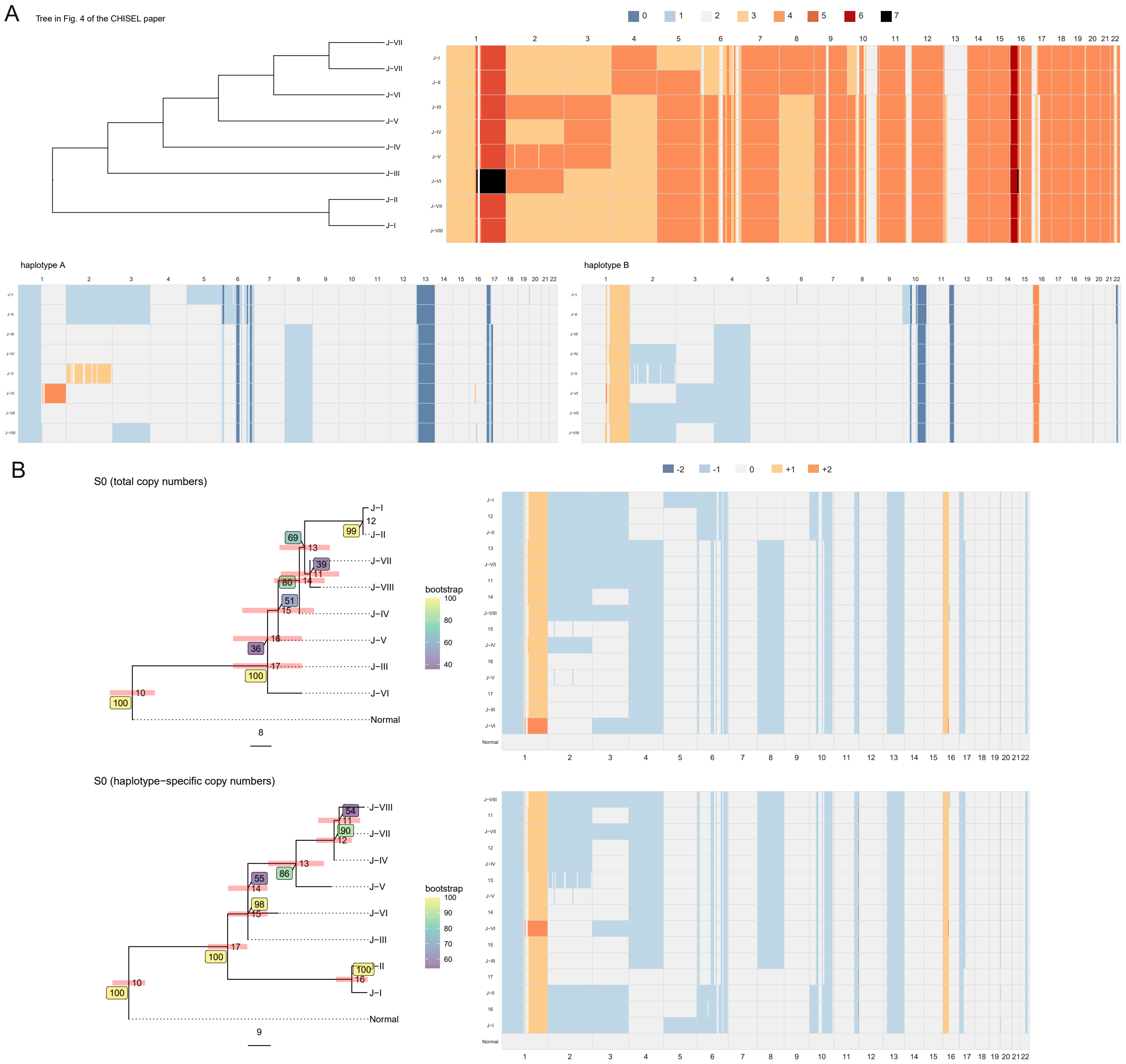

Fig. S19: The performance of CNETML on 8 tumour clones detected from 10,202 cells from all five sections of patient S0. **A**: The phylogenetic tree and heatmaps of copy number data based on Fig. 4 in [1]. **B**: The phylogenetic trees reconstructed by CNETML on relative total and haplotype-specific copy numbers, respectively. The interpretations of the trees in **B** are the same as those in Fig. S18. When using relative total copy numbers as input, the tree inferred by CNETML has a very different topology from the tree in [1]. But the separation of clone J-V and J-IV to the other clones have very low bootstrap support values (36% and 51%) respectively, suggesting the lack of information in the data, as clone J-V is separated due to chr2.A dup and clone J-IV is separated due to lack of mirrored-subclonal CNAs relative to clone J-V, J-VI, J-VII, and J-VIII in [1]. Clone J-VI is separated from all the other clones by CNETML with certainty probably due to the unique duplication on chr1q. The separation of clone J-1 and J-II with the other clones also has quite low bootstrap support (69%), which are separated from the other clones mainly by chr4 del, chr8 del, chr2.A del, chr3.A del, chr6p del, and chr10p del in [1]. Therefore, the lack of haplotype-specific information may cause the large uncertainties in the tree built by CNETML. When using relative haplotype-specific copy numbers as input, the tree inferred by CNETML is very similar to the tree in [1], except that the position of clone J-VI and J-IV are switched, which is probably because of the unique duplication on chr1q of clone J-VI. The trees were reconstructed in a similar approach to that in Fig. 7.

A

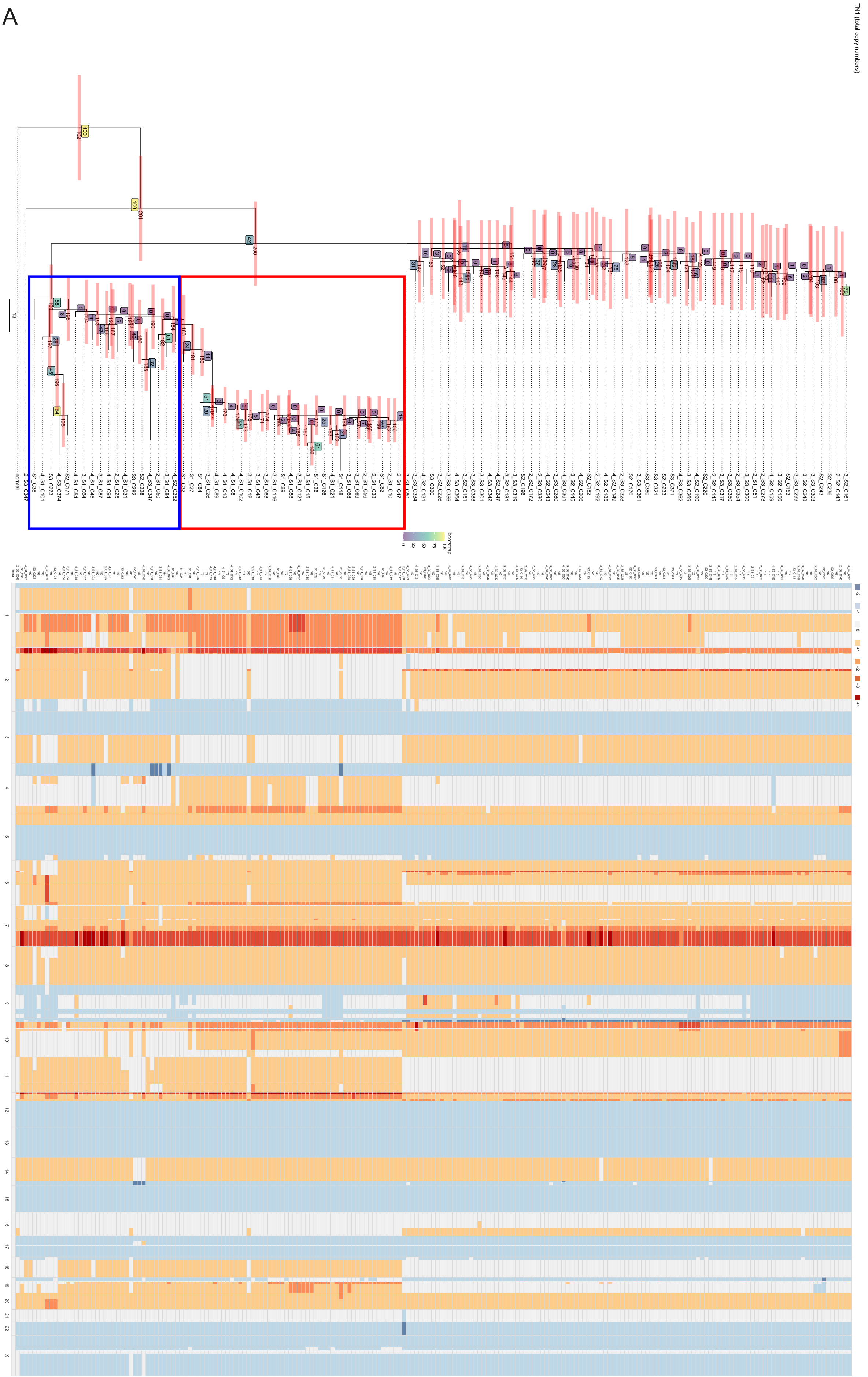

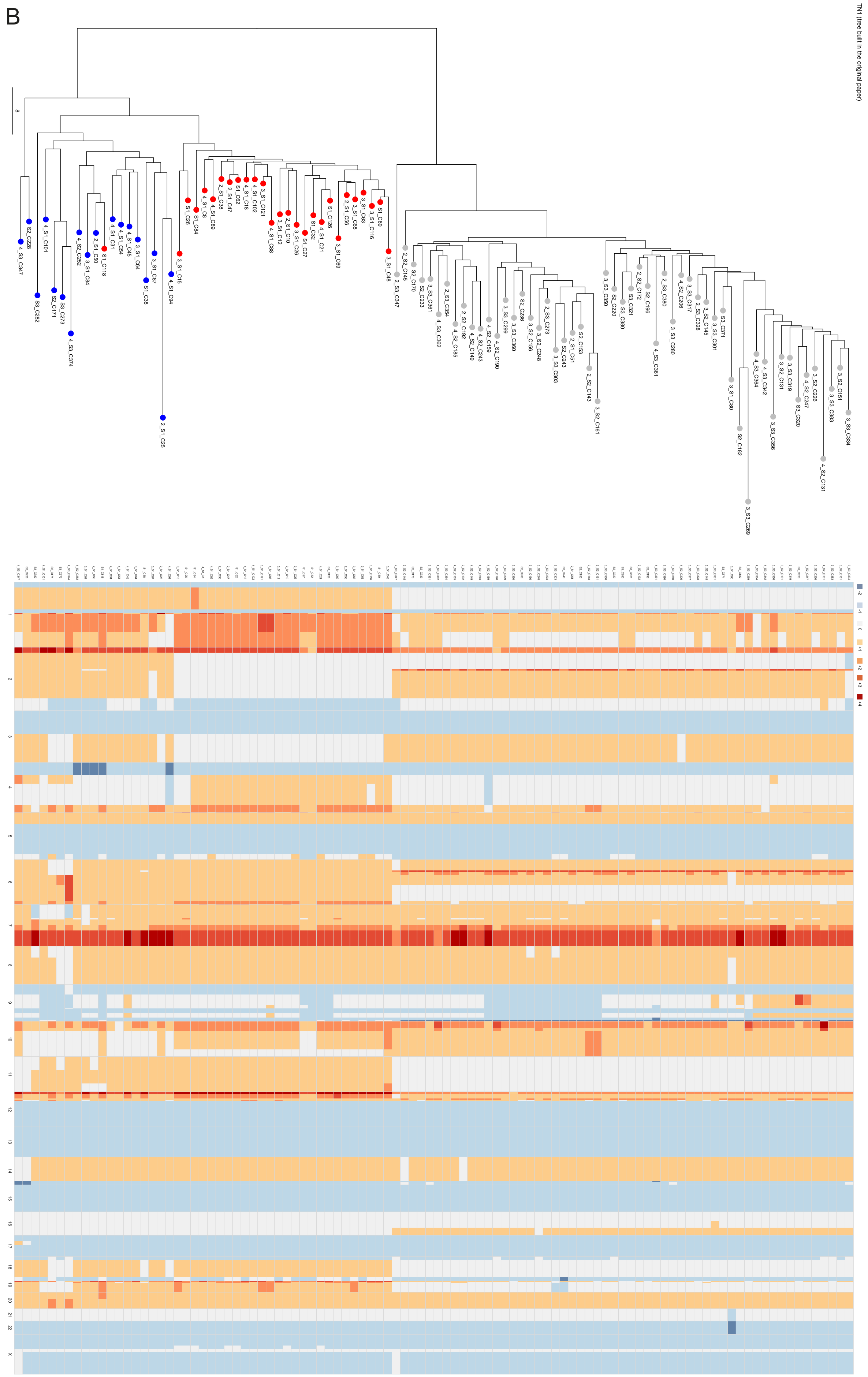
B
TN1 (tree built in the original paper)

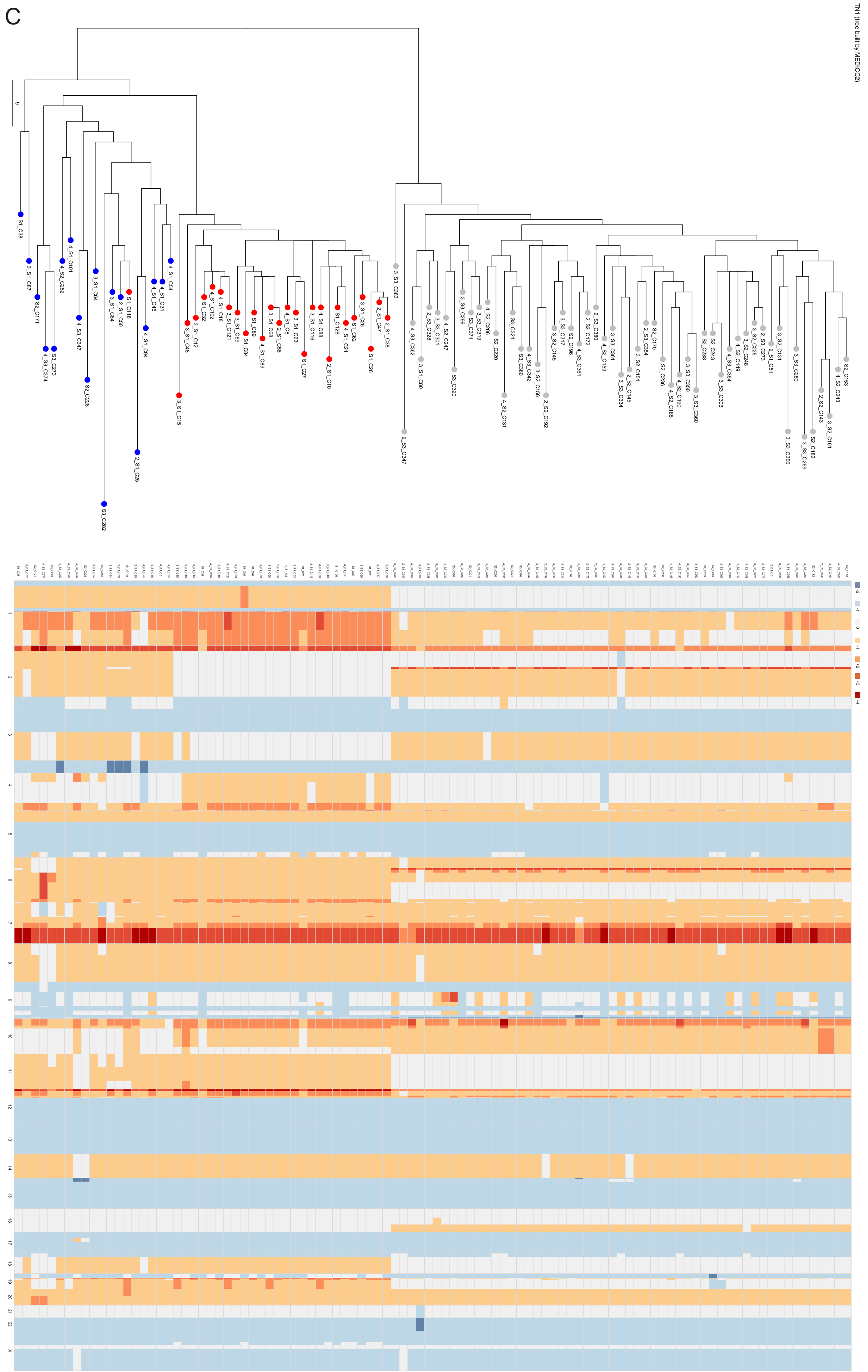
C
TN1 (tree built by MEDICC2)

Fig. S20: The performance of CNETML on 100 cells sampled from 1,100 cells of patient TN1. **A**: The phylogenetic tree reconstructed by CNETML and the heatmap of relative total copy number data ordered by both tip and internal nodes in the tree. **B**: The phylogenetic tree reconstructed by a balanced minimum evolution algorithm in Fig. 3 of the original paper reporting the single cell data [2] and the heatmap of relative total copy number data ordered by tip nodes in the tree, obtained by extracting the 100 sampled cells from the full tree in Fig. S23b of [3]. **C**: The phylogenetic tree reconstructed by MEDICC2 [3] and the heatmap of relative total copy number data ordered by tip nodes in the tree, obtained by extracting the 100 sampled cells from the full tree in Fig. S23a of [3]. We selected three groups of cells (indicated by red, blue, and no box in **A**) on the tree built by CNETML according to the tree topology, which are roughly distinguished by copy number alterations on chr1, chr2, chr3q, chr4q, chr6, chr10, chr11, chr16q, chr18, and chr19. The other two trees show three very similar groups of cells (indicated by red, blue, and grey dots in **B** and **C**), except that cell S1_C118 is grouped differently in **B** and **C**. The tree reconstructed by CNETML was obtained with a similar approach to that in Fig. 7, with the same interpretations as those reconstructed by CNETML in Fig. S18.

A

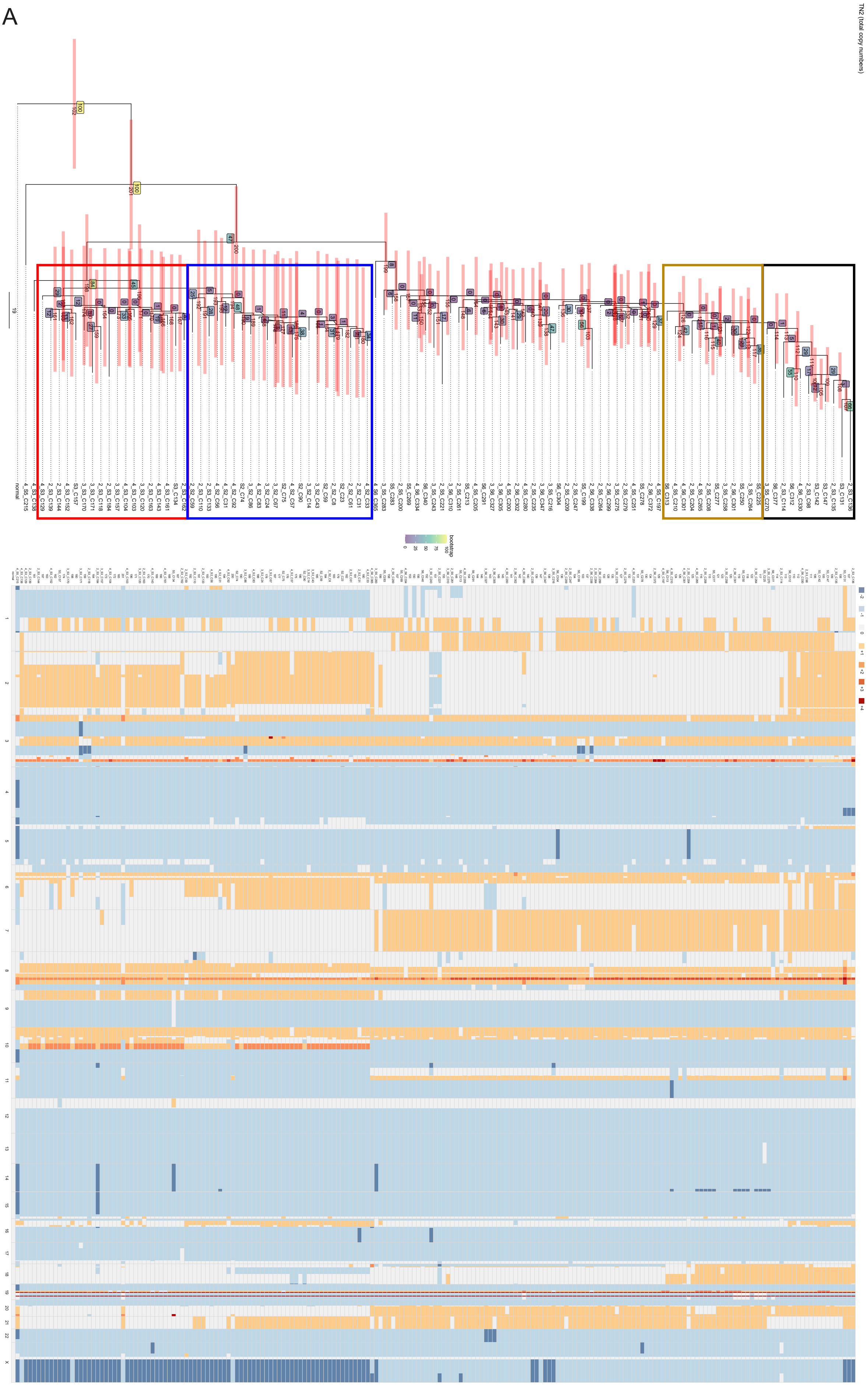

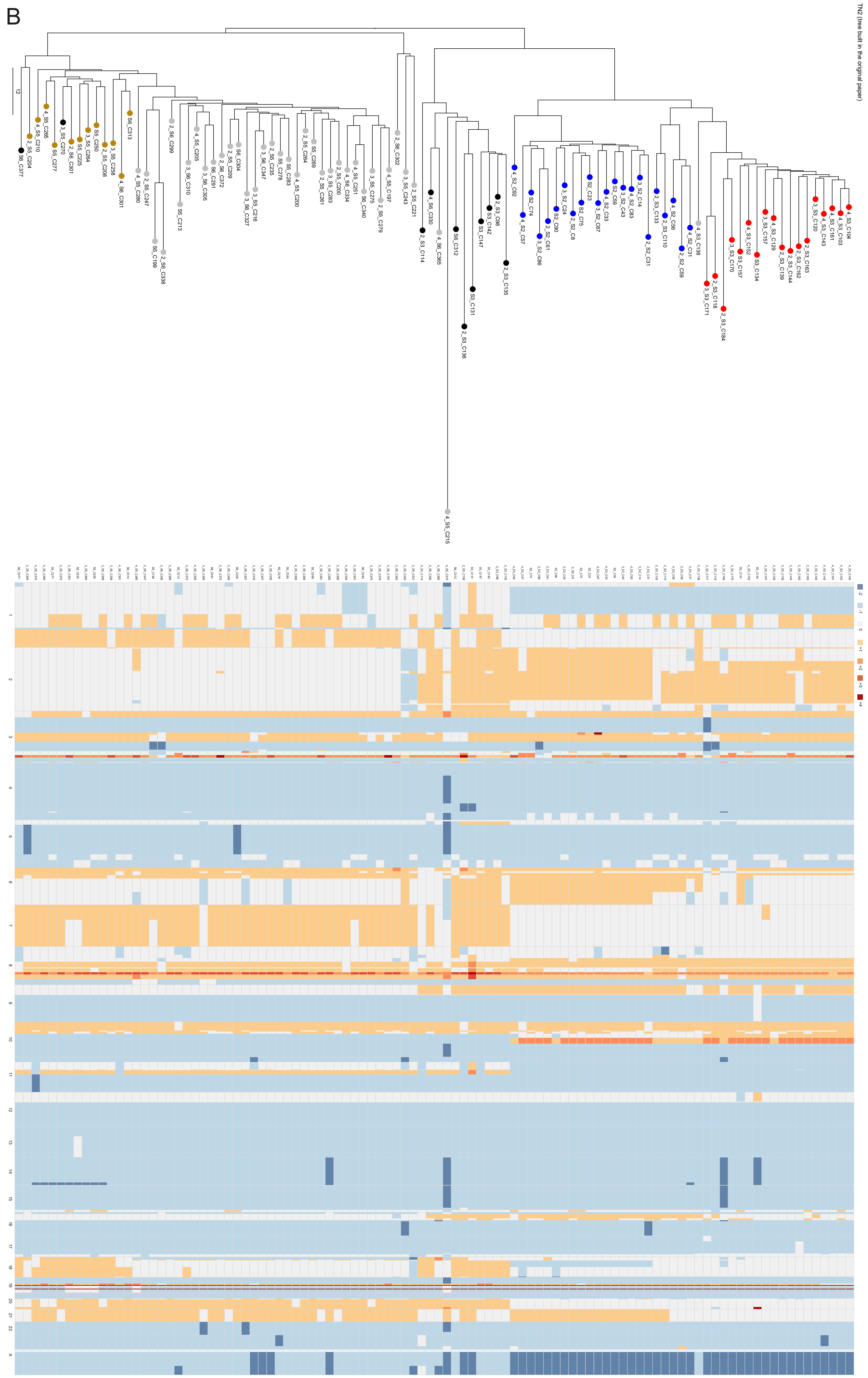
B
TN2 (tree built in the original paper)

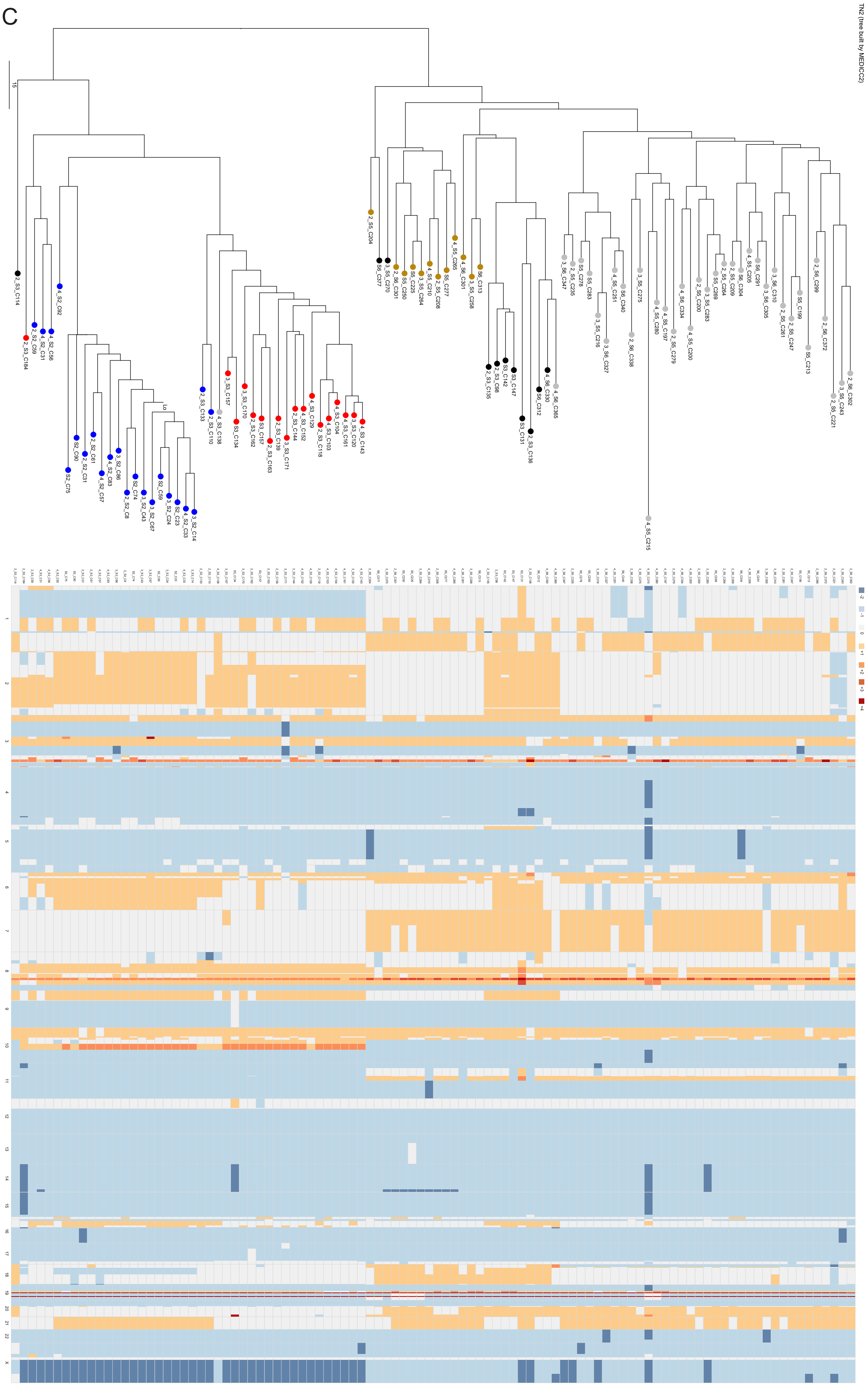
C
TN2 (tree built by MEDICC2)

Fig. S21: The performance of CNETML on 100 cells sampled from 1,023 cells of patient TN2. **A**: The phylogenetic tree reconstructed by CNETML and the heatmap of relative total copy number data ordered by both tip and internal nodes in the tree. **B**: The phylogenetic tree reconstructed by a balanced minimum evolution algorithm in Fig. 3 of the original paper reporting the single cell data [2] and the heatmap of relative total copy number data ordered by tip nodes in the tree, obtained by extracting the 100 sampled cells from the full tree in Fig. S23c of [3]. **C**: The phylogenetic tree reconstructed by MEDICC2 [3] and the heatmap of relative total copy number data ordered by tip nodes in the tree, obtained by extracting the 100 sampled cells from the full tree in Fig. 5 of [3]. We selected five groups of cells (indicated by red, blue, darkgoldenrod, black, and no box in **A**) on the tree built by CNETML according to the tree topology, which are roughly distinguished by copy number alterations on chr1, chr2, chr6, chr7, chr9p, chr11p, chr16p, chr18, chr20, chr21, and chrX. The other two trees show five similar groups of cells (indicated by red, blue, darkgoldenrod, black, and grey dots in **B** and **C**). Only the groupings of several cells are different from those in **A**, including cell S6_C377, 3_S5_C270, 4_S6_C365, and 4_S3_C138 in **B** and **C**, and cell 2_S3_C114 and 2_S3_C184 in **C**. The tree reconstructed by CNETML was obtained with a similar approach to that in Fig. 7, with the same interpretations as those reconstructed by CNETML in Fig. S18.

References

[1] Simone Zaccaria and Benjamin J Raphael. Characterizing allele- and haplotype-specific copy numbers in single cells with CHISEL. *Nature Biotechnology*, 39(2):207–214, 2021.

[2] Darlan C Minussi, Michael D Nicholson, Hanghui Ye, Alexander Davis, Kaile Wang, Toby Baker, Maxime Tarabichi, Emi Sei, Haowei Du, Mashiat Rabbani, Cheng Peng, Min Hu, Shanshan Bai, Yu-wei Lin, Aislyn Schalck, Asha Multani, Jin Ma, Thomas O. McDonald, Anna Casasent, Angelica Barrera, Hui Chen, Bora Lim, Banu Arun, Funda Meric-Bernstam, Peter Van Loo, Franziska Michor, and Nicholas E. Navin. Breast tumours maintain a reservoir of subclonal diversity during expansion. *Nature*, 592(7853):302–308, 2021.

[3] Tom L. Kaufmann, Marina Petkovic, Thomas B. K. Watkins, Emma C. Colliver, Sofya Laskina, Nisha Thapa, Darlan C. Minussi, Nicholas Navin, Charles Swanton, Peter Van Loo, Kerstin Haase, Maxime Tarabichi, and Roland F. Schwarz. MEDICC2: whole-genome doubling aware copy-number phylogenies for cancer evolution. *Genome Biology*, 23:241, 2022.
