# Supplementary material for: CNETML: maximum likelihood inference of phylogeny from copy number profiles of multiple samples
Source: Genome Biol. 2023 Jun 20;24:144. doi: 10.1186/s13059-023-02983-0 (PMC10283241; doi:10.1186/s13059-023-02983-0)
Supplement: Supplementary file 4 — Additional file 4: Review history. [file 13059_2023_2983_MOESM4_ESM.docx]

Review History

**First round of review**

**Reviewer 1**

**Were you able to assess all statistics in the manuscript, including the appropriateness of statistical tests used?**

**Yes:** It is a method paper. The statistics are new and I have questions in the main text.

**Were you able to directly test the methods?**

No

**Comments to author:**

The authors describe a maximum-likelihood-based approach to infer tree topology, node ages, and mutation rates from multi-sample and multi-timepoint low-coverage whole-genome bulk sequencing data. The method relies on estimates of relative total copy number, only.

The paper addresses a special case when multi-sample and multi-time data are available. While the idea of estimating mutation rates and node age is interesting, some serious issues remain that should be addressed in full.

Major issues

Relevance:
Some of the requirements and limitations of the approach should be summarized more clearly. Are there many multi-timepoint samples available? The general applicability of the approach will depend on how widely available these data are (or can reasonably be expected to be in the future), else it might be a very niche application.

Related to this question, how many time points, and samples are required? I assume mutation rates cannot be estimated if there is only one timepoint available. This is mentioned somewhat ambiguously at one point (multi-sample and/or multi-timepoint samples) but should be clearly communicated. Can the authors be a bit more specific about the number and time intervals between samples that would be required to obtain reasonable estimates? Is it possible to infer timings from multi-sample data sampled at one timepoint?

Clarity
The authors write: "Therefore, although a site is not as well defined as when modelling SNVs on individual nucleotides, it is feasible to consider a bin or a segment as a site."
Can you be clear about what the ramification of choosing site vs segment is for your algorithm? Are you using an input similar to MEDICC where sites are defined via consecutive bins with differences across samples? How would your algorithm's performance change if we use bins vs segments?

Is performance for fewer sites significantly worse? Figure 4 only shows results for 1000 and 10000 sites, Figure 3 includes up to 100 sites.

In practice, with related samples from one individual, I would not expect more than 200-300 sites (defined as varying segments). At 500kB resolution, the maximum number of bins is about 5000 for a human genome. What is the empirical range of sites in your data, given you only have a few samples at most, and only positions that vary across samples should be informative?

Mutation rates: Do you consider different mutation rates for different types of events? If not, why not? Copy number changes can be caused by a variety of mutational processes, which are biologically very distinct and differently regulated, it might be unreasonable to assume common mutation rates.

Limits of applicability
We have some questions about several of the authors' statements regarding the applicability of the method:

The authors write: "In summary, it seems entirely feasible to recover the phylogeny directly from relative copy numbers, such as those from QDNAseq, when WGD is not clonal."
However, WGD is often an early event and we would thus assume most of them will be clonal, which would limit the applicability of the approach. Can the authors be more quantitative about the percentage of samples or cancer types they think their method will be applicable in (using for example TCGA or PCAWG as background).

The authors write "However, when the mean duplication/deletion size was 5 Mbp, shorter than the typical focal CNA size, the bias was not very large, and the tree topologies were still recovered well"
In practice, we see much smaller focal CNA events, 5 Mbp seems like a rather large event. So again, this would indicate that the method doesn't work in many cases.

Minor issues
Second paragraph in introduction: "Given different input data and aims,..."
The separation into 4 groups is not clear to me. This needs to be elaborated on. In particular, I don't see the difference between 3 and 4, in case the sample is a cell.

Figure 3: description is insufficient, A, B, C don't depict accuracy based on the y-axis
Same for Figure 4.

"These distances were computed with function treedist in R library phangorn"
Can you say what this distance is based on mathematically? Readers need some intuition without having to look up the function definition in a package of a programming language they might not use.

"The rates and LUCA age were slightly overestimated with bin-level data on larger CNAs, which is probably due to larger overdispersion caused by dependencies between adjacent bins with the same copy numbers [56]."
I don't understand the rationale. Could you explain it differently?

Spelling (missestimated, osetosarcoma), please check

**Reviewer 2**

**Were you able to assess all statistics in the manuscript, including the appropriateness of statistical tests used?**

**Yes:** I believe the statistical methods as described are appropriate, with the possible exception of points noted in my comments to the Authors. I do not believe it needs further statistical review.

**Were you able to directly test the methods?**

No

**Comments to author:**

This paper describes CNETML, a new tool for phylogenetic inference of clonal lineage trees from copy number data, designed for use with shallow whole genome sequence (sWGS) data. The work seeks to address several limitations of current methods in this space, including work with total or relative copy number data, providing maximum likelihood inference, inferring edge lengths, and handling spatial and temporally disparate samples. The authors do a good job of summarizing relevant literature and explaining the advance of the present methods. The model here is complex and sophisticated, but with good justification in providing a comprehensive inference platform. The testing on simulated data provides good evidence the methods are effective, with refreshingly frank consideration for conditions under which they perform poorly. The application to real Barrett's esophagus patient data is similarly well handled, providing a compelling demonstration of the approach despite some limitations. Overall, I believe the work described is a valuable contribution to the literature that improves on the current tool set for the field in some important ways. While the methods have limitations, these are understandable and are generally well explored and acknowledged. My comments are therefore relatively minor, although there are some points I think could be explained or explored in a bit more depth.

1. The heuristic algorithm, using hill climbing with nearest neighbor interchange, would seem to have some risk of scaling poorly with the number of samples. While the empirical results look good, it would be worth seeing some stronger evidence about how performance scales with numbers of samples, or guidance to users about the limits of the scaling.

2. I would also expect that hill climbing might get stuck in local optima. Given that the authors have both exhaustive search and hill climbing implemented, it does prompt the question of how consistent they would be at least for some tree sizes where both approaches are practical.

3. It would also be helpful to see a bit more empirical justification for the assertion that one need not consider deconvolution for this kind of data. At least some of the literature on copy number variation would seem to suggest high subclonal heterogeneity is not unusual even for a single tumor site and time point. It would also be worth considering, either as a thought experiment or through empirical tests, what a user would see if there were subclonal heterogeneity in individual samples of this kind.

4. I do also question how effective will be the assumption when working with relative copy numbers of a baseline of 2^(N_WGD). It has commonly been observed that WGD is often followed by a cascade of more localized losses that leads to a true ploidy closer to three than four. Would this be a significant source of error for this method?

5. The question of errors in copy number profiles is only dealt with in the discussion and I am not convinced this is adequate given that calls from sWGS data can be expected to be noisy. It would seem straightforward to test how sensitive the method is to biases in sequencing or to errors in variant calls on simulated data at least. This could especially be an issue if there is subclonal copy number heterogeneity of the kind discussed in the previous point.

**Reviewer 3**

**Were you able to assess all statistics in the manuscript, including the appropriateness of statistical tests used?**

**Yes:** Appropriate as is.

**Were you able to directly test the methods?**

No

**Comments to author:**

This paper introduces CNETML, a method to infer a phylogeny given copy-number profiles of samples. CNETML is a maximum likelihood method where sites, corresponding to genomic bins or segments, change copy number based on a continuous-time non-reversible Markov chain. CNETML takes copy-number profiles as input, and seeks to infer a maximum likelihood phylogeny where each input profile corresponds to a leaf. CNETML is evaluated using simulated data and applied to two Barrett's esophagus tumors. While the paper is well written and of interest to the readership of this journal, I have concerns regarding scope and the experimental evaluation.

1. Scoping

Two comments regarding scoping of the work:

- Title mentions spatio-temporal samples. However, it is not clear if the method uses spatial information as a constraint.

- Why the focus on shallow WGS? While there is a description on how to process shallow WGS data, the method takes as input copy-number profiles (in fact, this is the starting point of the simulations). I do not understand the framing of CNETML as a method solely for shallow WGS. There are a lot of single-cell DNA sequencing data that can be analyzed using your method. For example, I'd be very interested in the performance of CNETML when given copy-number profiles inferred by CHISEL [1].

2. Experimental evaluation

- CNETML is based on the following assumptions: (1) independence of sites (no nested/overlapping CNAs), (2) sample homogeneity, each sample is composed of a single tumor clone, (3) input copy-number profiles are error free, and (4) constant duplication/deletion rates. Only violations to the first assumption are assessed. It would be good to assess violations to the other assumptions as well.

- How well does the heuristic perform relative to exhaustive enumeration? The paper states that exhaustive enumeration is feasible up to 7 samples. So one can run both the heuristic and the exact version of CNETML on such data, and assess decrease in likelihood/performance due to the heuristic.

- How well does L-BFGS-B infer model parameters when given the right tree topology?

- The introduction mention several methods for copy-number tree inference. However, the experimental evaluation does not include these methods (with the exception of MEDICC 2 for a restricted scenerio). Please benchmark against other methods.


Minor comments:

- What are relative CNPs? Relative to the most frequent copy-number value?

- Page 10, line 25. consistent => statistically consistent.

- Page 10, line 42. What are unique variant sites?

- Page 20, line 49. Interspersed how?

- Release simulated and real data to facilitate reproducibility and future benchmarking.

[1] Zaccaria, Simone, and Benjamin J. Raphael. "Characterizing Allele- and Haplotype-Specific Copy Numbers in Single Cells with CHISEL." Nature Biotechnology, September 2, 2020. <https://doi.org/10.1038/s41587-020-0661-6>.

**Authors Response**

**Point-by-point responses to the reviewers’ comments:**

**Reviewer #1:** The authors describe a maximum-likelihood-based approach to infer tree topology, node ages, and mutation rates from multi-sample and multi-timepoint low-coverage whole-genome bulk sequencing data. The method relies on estimates of relative total copy number, only.

The paper addresses a special case when multi-sample and multi-time data are available. While the idea of estimating mutation rates and node age is interesting, some serious issues remain that should be addressed in full.

Major issues

Relevance:
Some of the requirements and limitations of the approach should be summarized more clearly. Are there many multi-timepoint samples available? The general applicability of the approach will depend on how widely available these data are (or can reasonably be expected to be in the future), else it might be a very niche application.

Response:
The multi-timepoint shallow whole genome sequencing (sWGS) samples are common in the surveillance of Barrett’s esophagus which typically has a pre-malignant phase before diagnosis, where 777 samples were obtained from 88 patients and some of these samples were taken over a period of up to 15 years (https://doi.org/10.1038/s41591-020-1033-y). These types of samples can also be obtained for patients with inflammatory bowel disease (IBD) who may go through low-grade dysplasia and high-grade dysplasia before getting colitis-associated colorectal cancer (CA-CRC) and undergo surgery to remove CA-CRC. For example, 81 samples from 19 patients with IBD were obtained in (http://dx.doi.org/10.1136/gutjnl-2018-316191) and more such samples have been generated and under analysis (personal communication).
There are also multi-timepoint sWGS data from experimental evolution of organoids which was developed to study the process of tumorigenesis (https://doi.org/10.1101/2022.04.09.487529).
The recent development of non-invasive liquid biopsy approaches (https://doi.org/10.1038/s41571-022-00660-y) makes it relatively easier to get cell-free DNA (cfDNA) samples at multiple time points throughout the disease progression for longitudinal monitoring. For example, Liu et al. collected 476 serial samples from 123 patients with medulloblastoma and sequenced the isolated cfDNA with sWGS to assess measurable residual disease (https://doi.org/10.1016/j.ccell.2021.09.012). Boons et al. performed sWGS and copy number analysis of 195 cfDNA longitudinal samples taken from 43 patients with neuroendocrine neoplasms to explore the usage of cfDNA copy number alteration analysis as biomarker for diagnosis, prognosis, and follow-up (https://doi.org/10.1158/1078-0432.CCR-21-2291).
The recent human tumour atlas network (HTAN) aims to generate multiparametric spatio-temporal data of cancers during their evolution from precancerous lesions to advanced disease, including longitudinal sampling of pre-cancers, tumours, metastases, and treatment responses. In HTAN, there are currently 11 atlases across 46 organs of 36 caner types, among which publications are now available for 3 studies and the others are coming soon. In the study “An omic and multidimensional spatial atlas from serial biopsies of an evolving metastatic breast cancer” (https://doi.org/10.1016/j.xcrm.2022.100525), sWGS was performed along with other types of sequencing to characterize four serial biopsies of an individual with metastatic breast cancer during 3.5 years of therapy.
We have briefly incorporated the text above in the Background to better motivate the study. We also want to emphasize that CNETML is applicable to samples taken at a single time point, for which the mutation rates and node ages cannot be estimated. Therefore, CNETML provides a general framework for maximum likelihood inference of tumour phylogeny from copy number data. The capability of CNETML to use sampling timing information when available to estimate mutation rates and node ages from total copy numbers are novel. Therefore, we highlighted this in the title by specifying “spatio-temporal samples”. To avoid misunderstanding of the general applicability of our method, we have changed it to be “multiple samples”.

Related to this question, how many time points, and samples are required? I assume mutation rates cannot be estimated if there is only one timepoint available. This is mentioned somewhat ambiguously at one point (multi-sample and/or multi-timepoint samples) but should be clearly communicated. Can the authors be a bit more specific about the number and time intervals between samples that would be required to obtain reasonable estimates? Is it possible to infer timings from multi-sample data sampled at one timepoint?

Response:
Yes, the mutation rate can only be estimated with at least two time points. The branch length in the phylogenetic tree represents the amount of evolution which is the product of mutation rate and time. It is not possible to infer timings from multi-sample data at one time point, because the model is unidentifiable when there is no information on sampling times. Note that we can still infer phylogenies on data at one time point, but the branch lengths have different meanings (not the time but the amount of copy number alterations).
To obtain reasonable estimates, we need at least three samples and at least two time points. As shown in Figure 3 and Supplementary Figure 7, reasonable estimates can be obtained even when the time intervals among samples are small (between around 5 months to 3 years, Supplementary Table 5).
We have clarified these points in Background, subsection “Overview of CNETML”, and Discussion of the manuscript.

Clarity
The authors write: "Therefore, although a site is not as well defined as when modelling SNVs on individual nucleotides, it is feasible to consider a bin or a segment as a site." Can you be clear about what the ramification of choosing site vs segment is for your algorithm? Are you using an input similar to MEDICC where sites are defined via consecutive bins with differences across samples? How would your algorithm's performance change if we use bins vs segments?

Response:
The input to our algorithm is a copy number matrix, where each column is the copy number at a site and the sites are aligned across all samples. When using bins, they are regions of fixed size (e.g. 500 Kbp) and the consecutive bins with the same copy number are not merged. When using segments, they are regions of varying size which are obtained by joint segmentation across samples, namely merging the consecutive bins with the same copy number in a single sample until a change point which is defined across all samples. This is similar to the input of MEDICC. We have improved the definition of segments to avoid confusions.
Regarding the ramification of choosing site vs segment for our algorithm or our algorithm's performance when using bins or segments, we actually mentioned that when we discussed the performance of our algorithm under violation of independent sites assumption. As shown in Figure 6 and stated in the subsection “Performance under violation of independent sites assumption”, “the differences of using bin-level and segment-level data were small because the site patterns in the input data were similar in both cases, except that bin-level data might contain more sites and a larger number of the same pattern which lead to longer computing time. …… we recommend using segment-level data for faster computation, less correlation between segments, and better interpretability in practice.” We have clarified this point by adding “We will discuss the ramifications of using bin or segment as a site in Results”.

Is performance for fewer sites significantly worse? Figure 4 only shows results for 1000 and 10000 sites, Figure 3 includes up to 100 sites. In practice, with related samples from one individual, I would not expect more than 200-300 sites (defined as varying segments). At 500kB resolution, the maximum number of bins is about 5000 for a human genome. What is the empirical range of sites in your data, given you only have a few samples at most, and only positions that vary across samples should be informative?

Response:
The number of sites in Figure 3 and 4 is the size of the genome or the total number of sites on a genome. The numbers of varying sites, or variant sites as defined in subsection “Overview of CNETML” in the manuscript, depend on the mutation rates in the simulation. The number of unique variant sites, sites with unique site patterns, is more informative for phylogeny reconstruction.
We counted the number of (unique) variant sites, in our simulated data in Figure 3 and included the results as Supplementary Figure 2 and Supplementary Table 3. There are only between 90 and 180 variant sites when we simulated 1000 sites at mutation rate 0.001 per haplotype per site per year, yet the topologies of 82 out of 100 trees have been correctly recovered with the total copy numbers, as suggested by the values of normalized RF distance in Figure 3. Therefore, the performance of CNETML should be good with 200-300 variant sites/segments in practice. We also included the numbers of reconstructed trees with correct topologies in Supplementary Table 3 for reference. We have incorporated these results into subsection “Performance on reconstructing trees and ancestral state” of the manuscript.
We showed the results of 10000 sites to demonstrate the method is statistically consistent with a large number of sites. We did not include 100 sites in Figure 4 as the plots will be too crowded and the results were expected to be less accurate.

Mutation rates: Do you consider different mutation rates for different types of events? If not, why not? Copy number changes can be caused by a variety of mutational processes, which are biologically very distinct and differently regulated, it might be unreasonable to assume common mutation rates.

Response:
In this manuscript, we only consider focal/site duplication and deletion rates. This is a practical first step towards estimating copy number alteration rate by calendar time, as we often consider copy number gain/loss in terms of duplication and deletion which include the majority of copy number alterations (https://doi.org/10.1038/ng.2760). We agree that there are a number of potential mutational processes causing copy number changes and these processes may occur at varying rates. However, disentangling their different effects and relative rates will require much higher depth sequencing than the data considered here. Our approach can be expanded to incorporate chromosomal gain/loss and WGD in the model, but the combinatorial complexity is challenging to overcome and is for further work.
Regarding the assumption of constant mutation rates, it is true that the rates are very likely to change during tumour growth. However, there are few studies on the rates of copy number alterations and how they change. The assumption of constant rate is a reasonable trade-off of model complexity and biological reality for now. It allows us to get approximate timing information of early copy number alteration events, which may be helpful in estimating the potential onset age of the disease for a patient. The confidence intervals obtained from bootstrapping can reflect the uncertainties of the estimations to some extent. It is entirely possible to allow rate variation with a random local clock as in the Bayesian analysis approach (https://doi.org/10.1038/s41467-017-02621-x). However, if we model rate variation in the current maximum likelihood framework, there will be too many parameters and insufficient information in the data to get reliable estimates. We consider the incorporation of rate variation in a Bayesian framework as future work too.
These points have been briefly incorporated into subsection “Overview of CNETML” and Discussion of the manuscript.

Limits of applicability
We have some questions about several of the authors' statements regarding the applicability of the method:

The authors write: "In summary, it seems entirely feasible to recover the phylogeny directly from relative copy numbers, such as those from QDNAseq, when WGD is not clonal."
However, WGD is often an early event and we would thus assume most of them will be clonal, which would limit the applicability of the approach. Can the authors be more quantitative about the percentage of samples or cancer types they think their method will be applicable in (using for example TCGA or PCAWG as background).

Response:
We agree that WGD often occurs early in cancer development, but the clonality of WGD depends on the properties of available samples from patients such as sampling times. The longitudinal samples obtained may contain pre-cancer samples which are very unlikely to have WGD. In previous large tumour programs such as TCGA (https://doi.org/10.1016/j.cell.2018.03.022) and PCAWG (https://doi.org/10.1038/s41586-020-1969-6), most donors have one primary sample and only a few donors have multiple tumour samples. The known timing of WGD events in the 2,658 cancers from PCAWG has an intermediate ranking among the somatic driver events and there are only around 38% samples with WGD among the 60 colorectal adenocarcinoma samples (https://doi.org/10.1038/s41586-019-1907-7). Bielski et al. found WGD in around 30% of 9,692 advanced cancer patients, varying between 58% to less than 5% (https://doi.org/10.1038/s41588-018-0165-1). This suggests that a large proportion of cancer samples do not have WGDs.
In our initial test, we used a similar approach to that adopted in the paper from Martinez et al. (http://dx.doi.org/10.1038/s41467-017-02621-x) which was applied on copy number data called from SNP array, but it turns out that the ways of scaling from absolute to relative copy numbers affect the performance of tree building and the previous random rounding approach is not appropriate in our case (Supplementary Figure 11). We have rerun simulations to use direct rounding to the nearest integer and the results suggest that CNETML inferred most phylogenies well on the simulated datasets with clonal WGDs, similar to MEDICC2 (Supplementary Figure 12). To be consistent with applications on real data where the numbers of WGDs are often unknown, we also tested using the rounded mean copy number as the baseline to scale the absolute copy numbers in the simulated data. The results were similar to those obtained by using the baseline based on the number of WGDs (updated Figure 5). We have used the rounded mean copy number as the baseline and direct rounding on haplotype-specific copy numbers detected from single-cell data of a breast cancer patient which have clonal WGDs (https://doi.org/10.1038/s41587-020-0661-6). The results (Supplementary Figure 17 and 18) suggest that CNETML reconstructed phylogenies largely consistent with the previous inferences and the uncertainties in the inference due to the lack of information were well reflected by the bootstrap support values. Therefore, CNETML is widely applicable to samples with unknown WGD status which have relative copy numbers or absolute copy numbers which are scaled appropriately.
These results have been incorporated into subsection “Performance on relative copy numbers” and Discussion of the manuscript.

The authors write "However, when the mean duplication/deletion size was 5 Mbp, shorter than the typical focal CNA size, the bias was not very large, and the tree topologies were still recovered well"
In practice, we see much smaller focal CNA events, 5 Mbp seems like a rather large event. So again, this would indicate that the method doesn't work in many cases.

Response:
We are sorry that the text should be “longer than the typical focal CNA size”. As stated in the prior paragraph, “focal CNAs are typically defined as CNAs of size no larger than 3 Mbp”. The performances of CNETML are actually better with smaller size. We showed results in Figure 6 when the mean size is 500 Kbp, the simulated trees had smaller distances to real trees and the differences of estimated node ages and mutation rates were also smaller.

Minor issues
Second paragraph in introduction: "Given different input data and aims,..." The separation into 4 groups is not clear to me. This needs to be elaborated on. In particular, I don't see the difference between 3 and 4, in case the sample is a cell.

Response:
We distinguish bulk sequencing sample and single cell sequencing sample, because different properties of the data represent different meanings and bring different challenges in phylogeny inference.
A bulk sample may be affected by intratumour heterogeneity and consist of heterogeneous subpopulations of cells. Therefore, the phylogeny of bulk samples has different interpretations than the phylogeny of single cells which explicitly reflects the cell lineages.
In addition, single-cell sequencing data may include hundreds to thousands of individual cells at a time whereas bulk-sequencing data often include much fewer samples. Therefore, scalability is a critical factor in designing a new phylogeny inference method for data generated from these different types of platforms.
To reduce the ambiguities, we have improved the description to be “single cell tree” and “bulk sample tree”. We also acknowledge that some methods are applicable for both types of data, such as MEDICC2.

Figure 3: description is insufficient, A, B, C don't depict accuracy based on the y-axis
Same for Figure 4.
Response:
The measurements on the y-axis in Figure 3 and 4 imply the accuracy. We have improved the descriptions by specifying the metrics used for measuring the accuracy.

"These distances were computed with function treedist in R library phangorn" Can you say what this distance is based on mathematically? Readers need some intuition without having to look up the function definition in a package of a programming language they might not use.
Response:
We did not include the definition of those distances to simplify the descriptions. To give readers more intuitions of these distances and how they relate to the accuracy of tree building, we have incorporated distance definitions.

"The rates and LUCA age were slightly overestimated with bin-level data on larger CNAs, which is probably due to larger overdispersion caused by dependencies between adjacent bins with the same copy numbers [56]."
I don't understand the rationale. Could you explain it differently?
Response:
When the CNAs have larger size, there are dependencies between more adjacent bins with the same copy number. These dependencies may cause larger variance of the number of changes. Overdispersion occurs when the variance of the number of changes exceeds the mean under the assumption of a Poisson distribution of the number of mutations. To make it clear, we have changed the sentence to be “The estimated rates and LUCA age were slightly higher with larger variances for bin-level data on larger CNAs, which is probably caused by dependencies between adjacent bins with the same copy numbers”.

Spelling (missestimated, osetosarcoma), please check
Response:
We have corrected the spelling.


**Reviewer #2:** This paper describes CNETML, a new tool for phylogenetic inference of clonal lineage trees from copy number data, designed for use with shallow whole genome sequence (sWGS) data. The work seeks to address several limitations of current methods in this space, including work with total or relative copy number data, providing maximum likelihood inference, inferring edge lengths, and handling spatial and temporally disparate samples. The authors do a good job of summarizing relevant literature and explaining the advance of the present methods. The model here is complex and sophisticated, but with good justification in providing a comprehensive inference platform. The testing on simulated data provides good evidence the methods are effective, with refreshingly frank consideration for conditions under which they perform poorly. The application to real Barrett's esophagus patient data is similarly well handled, providing a compelling demonstration of the approach despite some limitations. Overall, I believe the work described is a valuable contribution to the literature that improves on the current tool set for the field in some important ways. While the methods have limitations, these are understandable and are generally well explored and acknowledged. My comments are therefore relatively minor, although there are some points I think could be explained or explored in a bit more depth.

1. The heuristic algorithm, using hill climbing with nearest neighbor interchange, would seem to have some risk of scaling poorly with the number of samples. While the empirical results look good, it would be worth seeing some stronger evidence about how performance scales with numbers of samples, or guidance to users about the limits of the scaling.

Response:
Previously, we tested the performance of stochastic tree search algorithm on simulated data with 10 to 30 samples, as we thought 30 is a large number for bulk-sequencing samples from an individual patient. To get the limits of the scaling, we have tested the algorithm on 50, 100, and 200 samples as well. It took a long time (around 15h) for 100 samples. We performed tests on 200 samples with a time limit of 120h, 85 out of 100 cases finished when mutation rate was 0.001 per haplotype per site per year, and 87 out of 100 cases finished when mutation rate was 0.01 per haplotype per site per year.
The results (Supplementary Figure 3) suggest that the distances of reconstructed trees to simulated true trees increased almost linearly with the number of samples, with the coefficient being around 0.01 for normalized RF distance, around 0.36 for branch score distance when mutation rate was high (0.01), and around 0.8 for branch score distance when mutation rate was low (0.001). Therefore, the topologies of reconstructed trees were less affected by the increasing number of samples whereas the branch lengths were more affected especially when there were fewer observed mutations. We have incorporated these results into subsection “Performance on reconstructing trees and ancestral state” of the manuscript.

2. I would also expect that hill climbing might get stuck in local optima. Given that the authors have both exhaustive search and hill climbing implemented, it does prompt the question of how consistent they would be at least for some tree sizes where both approaches are practical.

Response:
To check the consistency of exhaustive search and hill climbing algorithms, we have tested both algorithms on data with 5, 6, and 7 samples. As expected, the differences of the minimal negative log likelihood values between those obtained from the heuristic and exact version of CNETML increased with the number of samples (Supplementary Figure 4). However, the heuristic version of CNETML still had reasonably good performance. The distributions of the distances of the reconstructed trees to the true trees were very similar between the heuristic and exhaustive search version, with slightly larger differences from the heuristic version on the branch score distance (Supplementary Figure 5). Moreover, the heuristic version of CNETML reconstructed the same tree topologies as the exact version on data with 5 samples and around 70% of the same tree topologies on data with 7 samples (Supplementary Table 4). Therefore, the hill climbing algorithm in CNETML seems capable of escaping from local optima for a small number of samples. These results have been incorporated into subsection “Performance on reconstructing trees and ancestral states” of the manuscript.
Previously, we mentioned in the manuscript that the exhaustive search is for fewer than seven samples. This limitation was designed for a laptop in a couple of hours. Actually, the program could also finish in around 15h for data with seven samples in our tests on computer clusters. We have revised the description to say exhaustive search is efficient for trees of no more than seven samples in subsection “Overview of CNETML” of the manuscript.

3. It would also be helpful to see a bit more empirical justification for the assertion that one need not consider deconvolution for this kind of data. At least some of the literature on copy number variation would seem to suggest high subclonal heterogeneity is not unusual even for a single tumor site and time point. It would also be worth considering, either as a thought experiment or through empirical tests, what a user would see if there were subclonal heterogeneity in individual samples of this kind.

Response:
If the clonal deconvolution of copy number data is feasible, we recommend applying our method to build phylogenetic trees based on the detected clones. However, with low coverage sequencing data, it is hard to do reliable deconvolution.
To see how subclonal heterogeneity affects phylogeny inference with our method, we postprocessed the simulated data to get data with subclonal structure. We assumed the originally simulated data is for clones rather than samples. Then we mixed clones to get the data for each sample by specifying the fraction of an original clone (the clone with the same ID as the sample) f_d and sampling the fraction of the other n clones (including the normal clone) with uniform Dirichlet distribution which is then multiplied by 〖(1-f〗_d). We simulated data with f_d∈{1,0.8,0.6,0.5,0.4} and n∈{2,3}. The results (Supplementary Figure 15) suggest that CNETML performed well when the original clone was dominant with a larger fraction than the other clones in the sample when the samples were at the same time point, with no less than 70% of the tree topologies correctly recovered. Since CNETML performed similarly with a sample having either 3 or 4 clones, we then checked its performance on simulated samples at different time points (dt = 1 year) with each sample having 4 clones (Supplementary Figure 16). More than 55% of the tree topologies were still correctly recovered when the original clone was dominant. When the mutation rate was low, the estimated duplication and deletion rates were close to the true values when the original clone was dominant whereas the estimated LUCA age slightly increased with decreasing fraction of original clone. When the mutation rate was high, the estimated duplication and deletion rates were more affected by ITH and declined almost linearly with decreasing fraction of original clone whereas the LUCA age was generally underestimated. These results and the simulation approach have been incorporated into subsection “Performance under violation of model assumption” and Methods of the manuscript respectively.

4. I do also question how effective will be the assumption when working with relative copy numbers of a baseline of 2^(N_WGD). It has commonly been observed that WGD is often followed by a cascade of more localized losses that leads to a true ploidy closer to three than four. Would this be a significant source of error for this method?

Response:
The baseline of 2^(N_WGD) was used in simulations where there are no noise and copy number gain and loss are equally likely. We used the simulated data to show that CNETML can work on relative copy numbers. In real data, it is hard to know the accurate number of WGDs and hence the baseline should often be based on the rounded mean copy number.
To be consistent with real data, we tested the approach of converting simulated data to relative copy numbers based on the rounded mean copy number. With haplotype-specific copy number, we divided the observed copy number by the rounded mean copy number for each haplotype. With total copy number of the genome, we divided the observed copy number by the rounded half mean copy number. We also found the previous random rounding approach introduced large bias in data with clonal WGDs (Supplementary Figure 11). Therefore, we have revised the rounding approach to be direct rounding to the nearest integer. The results were similar when using either 2^(N_WGD) or the rounded mean copy number as the baseline and CNETML achieved good performance on datasets with different types of WGDs (updated Figure 5, Supplementary Figure 12).
We have used the rounded mean copy number as the baseline and direct rounding on haplotype-specific copy numbers detected from single-cell data of a breast cancer patient which have clonal WGDs (https://doi.org/10.1038/s41587-020-0661-6). The results (Supplementary Figure 17 and 18) suggest that CNETML reconstructed phylogenies largely consistent with the previous inferences from tumour clones detected from single cell data and the uncertainties in the inference due to the lack of information were well reflected by the bootstrap support values.
These results have been incorporated into subsection “Performance on relative copy numbers” and Discussion of the manuscript.

5. The question of errors in copy number profiles is only dealt with in the discussion and I am not convinced this is adequate given that calls from sWGS data can be expected to be noisy. It would seem straightforward to test how sensitive the method is to biases in sequencing or to errors in variant calls on simulated data at least. This could especially be an issue if there is subclonal copy number heterogeneity of the kind discussed in the previous point.

Response:
We agree that the real data is noisy which limits the applicability of the methods especially when errors and subclonal heterogeneity are not accounted for by other approaches. We have incorporated the simulation of data with different error rates of copy number calling, where errors may be caused by sequencing noise, sample purity, and ITH. We simulated data of 5 samples with increasing fraction of sites with error f_e∈{0,0.1,0.3,0.5,0.7}. When f_e is specified, we randomly selected 〖round(m*f〗_e) sites in the genome to change their copy numbers. We sampled the new copy number from a Poisson distribution with the mean being the original copy number at the site. If the new copy number is larger than the pre-specified maximum copy number c_max, we set it to c_max.
The results (Supplementary Figure 13) suggest that more than 70% of the tree topologies were correctly recovered with relatively small branch length estimation errors when the samples were at the same time point and the mutation rate was high, although the performance of CNETML generally decreased with the increasing error rates especially when the mutation rate was low. We further checked how CNETML performed when the samples were at different time points under high mutation rates (0.01 per haplotype per site per year) (Supplementary Figure 14). CNETML still correctly reconstructed no less than 70% of the tree topologies although the branch length estimation errors became larger. With increasing error rates, the estimated duplication rate and LUCA age decreased whereas the estimated deletion rate slightly increased. We have incorporated the simulation approach and results into subsection “Performance under violation of model assumption” and Discussion of the manuscript respectively.

**Reviewer #3**: This paper introduces CNETML, a method to infer a phylogeny given copy-number profiles of samples. CNETML is a maximum likelihood method where sites, corresponding to genomic bins or segments, change copy number based on a continuous-time non-reversible Markov chain. CNETML takes copy-number profiles as input, and seeks to infer a maximum likelihood phylogeny where each input profile corresponds to a leaf. CNETML is evaluated using simulated data and applied to two Barrett's esophagus tumors. While the paper is well written and of interest to the readership of this journal, I have concerns regarding scope and the experimental evaluation.

1. Scoping

Two comments regarding scoping of the work:

- Title mentions spatio-temporal samples. However, it is not clear if the method uses spatial information as a constraint.

Response:
We used the term “spatio” to refer to samples taken from multiple locations of a patient and did not use the spatial information in the inference. Because CNETML can also be applied to samples taken at a single time point, which is more available in practice, we have changed the title to be “multiple samples” to avoid misunderstanding of the general applicability of CNETML.

- Why the focus on shallow WGS? While there is a description on how to process shallow WGS data, the method takes as input copy-number profiles (in fact, this is the starting point of the simulations). I do not understand the framing of CNETML as a method solely for shallow WGS. There are a lot of single-cell DNA sequencing data that can be analyzed using your method. For example, I'd be very interested in the performance of CNETML when given copy-number profiles inferred by CHISEL [1].

Response:
The motivation of this method is the availability of shallow WGS data from multiple samples taken from different locations and time points of patients. When dealing with single-cell DNA sequencing data which typically include hundreds to thousands of cells, scalability is a critical issue. Our method was designed with bulk samples in mind and hence did not consider scalability. Therefore, currently CNETML can only be used on a small number of cells, or the clones detected from single cell data as those in the CHISEL paper. We want to emphasize that CNETML makes use of a Markov model of copy number evolution which enables the estimation of mutation rates on longitudinal shallow WGS data. The longitudinal shallow WGS data seems less expensive and more common for bulk DNA sequencing compared with single cell DNA sequencing. The model of copy number evolution makes CNETML applicable to haplotype-specific copy number data as well, which may be detected from deep sequencing data or with more advanced methods such as CHISEL. We agree that CNETML can actually be used to any integer total or haplotype-specific copy number profiles regardless of how they are detected from whatever the sequencing techniques, which was mentioned in Discussion.
Despite the caveats outline above, we have followed the reviewer’s suggestion and applied CNETML on data in Fig.3 and Fig.4 of the CHISEL paper. The data in Fig.3 include 6 clones (5 tumour clones and one diploid clone I) detected from 1,448 cells in section E of a breast cancer patient S0, which is denoted as dataset S0E subsequently. The data in Fig.4 include 8 tumour clones detected from 10,202 cells from all five sections of patient S0, which is denoted as dataset S0 subsequently. WGD was identified as a clonal event in nearly all tumour cells of patient S0. To mitigate the effect of WGD in the inference, we processed the data to get relative copy numbers by dividing the absolute copy numbers of each haplotype by the rounded mean value for that haplotype and rounding the resultant values to the nearest integers. Then we obtained the relative total copy numbers by adding up the relative copy numbers of each haplotype. We applied CNETML on both relative total copy numbers and relative haplotype-specific copy numbers (Supplementary Figure 17 and 18). The trees in the CHISEL paper were built based on the model of interval copy number alteration events and maximum parsimony principle. In Fig.3, Clone IV and V are at the internal nodes of a non-binary tree. To compare with the trees built by CNETML, we converted the tree in Fig.3 by creating a tip node for clone IV and V (Supplementary Figure 17).
For dataset S0E, when using relative total copy numbers as input, clone II in the tree inferred by CNETML has a different position from the tree in the CHISEL paper (bootstrap support 52%). When using relative haplotype-specific copy numbers as input, clone IV in the tree inferred by CNETML has a different position from the tree in the CHISEL paper (bootstrap support 40%). In the CHISEL paper, the events separating clone II (chr2.A del, chr3.A del) and the other clones (chr2.B del, chr3.B del) are mainly the mirrored-subclonal CNAs on chromosome 2 and 3. The overall consistency and uncertainty in placing clone II or clone IV (relative to clone II) suggest that CNETML can reconstruct reasonable phylogenies from tumour clones detected from single cell data.
For dataset S0, when using relative total copy numbers as input, the tree inferred by CNETML has a very different topology from the tree in the CHISEL paper. But the separation of clone J-V and J-IV to the other clones have very low bootstrap support values (36% and 51%) respectively suggesting the lack of information in the data, as clone J-V is separated due to chr2.A dup and clone J-IV is separated due to lack of mirrored-subclonal CNAs relative to clone J-V, J-VI, J-VII, and J-VIII in the CHISEL paper. Clone J-VI is separated from all the other clones by CNETML with certainty probably due to the unique duplication on chr1q. The separation of clone J-1 and J-II with the other clones also has quite low bootstrap support (69%), which are separated from the other clones mainly by chr4 del, chr8 del, chr2.A del, chr3.A del, chr6p del, and chr10p del in the CHISEL paper. Therefore, the lack of haplotype-specific information may cause the large uncertainties in the tree built by CNETML. When using relative haplotype-specific copy numbers as input, the tree inferred by CNETML is very similar to the tree in the CHISEL paper, except that the position of clone J-VI and J-IV are switched, which is probably because of the unique duplication on chr1q of clone J-VI.
These results have been briefly included in Discussion of the manuscript. The details of the results were included in the legends of Supplementary Figure 17 and 18.


2. Experimental evaluation

- CNETML is based on the following assumptions: (1) independence of sites (no nested/overlapping CNAs), (2) sample homogeneity, each sample is composed of a single tumor clone, (3) input copy-number profiles are error free, and (4) constant duplication/deletion rates. Only violations to the first assumption are assessed. It would be good to assess violations to the other assumptions as well.

Response:
To assess violation of assumption 2, we had added scripts to postprocess the data generated by our simulation program CNETS to get data with subclonal structure. We assumed the originally simulated data is for clones rather than samples. Then we mixed clones to get the data for each sample by specifying the fraction of an original clone (the clone with the same ID as the sample) f_d and sampling the fraction of the other n clones (including the normal clone) with uniform Dirichlet distribution which is then multiplied by 〖(1-f〗_d). We applied the above procedure on the simulated data with f_d∈{1,0.8,0.6,0.5,0.4} and n∈{2,3}. The results (Supplementary Figure 15) suggest that CNETML performed well when the original clone was dominant with a larger fraction than the other clones in the sample when the samples were at the same time point, with no less than 70% of the tree topologies correctly recovered. Since CNETML performed similarly with a sample having either 3 or 4 clones, we then checked its performance on simulated samples at different time points (dt = 1 year) with each sample having 4 clones (Supplementary Figure 16). More than 55% of the tree topologies were still correctly recovered when the original clone was dominant. When the mutation rate was low, the estimated duplication and deletion rates were close to the true values when the original clone was dominant whereas the estimated LUCA age slightly increased with decreasing fraction of original clone. When the mutation rate was high, the estimated duplication and deletion rates were more affected by ITH and declined almost linearly with decreasing fraction of original clone whereas the LUCA age was generally underestimated. These results and the simulation approach have been incorporated into subsection “Performance under violation of model assumption” and Methods of the manuscript respectively.
To assess violation of assumption 3, we have incorporated the simulation of data with different error rates of copy number calling in CNETS, where errors may be caused by sequencing noise, sample purity, and ITH. We simulated data of 5 samples with increasing fraction of sites with error f_e∈{0,0.1,0.3,0.5,0.7}. When f_e is specified, we randomly selected 〖round(m*f〗_e) sites in the genome to change their copy numbers. We sampled the new copy number from a Poisson distribution with the mean being the original copy number at the site. If the new copy number is larger than the pre-specified maximum copy number c_max, we set it to c_max. The results (Supplementary Figure 13) suggest that more than 70% of the tree topologies were correctly recovered with relatively small branch length estimation errors when the samples were at the same time point and the mutation rate was high, although the performance of CNETML generally decreased with the increasing error rates especially when the mutation rate was low. We further checked how CNETML performed when the samples were at different time points under high mutation rates (0.01 per haplotype per site per year) (Supplementary Figure 14). CNETML still correctly reconstructed no less than 70% of the tree topologies although the branch length estimation errors became larger. With increasing error rates, the estimated duplication rate and LUCA age decreased whereas the estimated deletion rate slightly increased. These results and the simulation approach have been incorporated into subsection “Performance under violation of model assumption” and Methods of the manuscript respectively.
Regarding assumption 4, it is expected that the estimation of node ages and duplication/deletion rates will become unreliable when the constant rate assumption is seriously violated. For the assessment, it seems feasible to revise CNETS to allow a different rate at each branch, but it is hard to choose appropriate parameters as there are few studies on the rates of copy number alterations and how they change. The constant rate assumption is a reasonable trade-off of model complexity and biological reality for now. It allows us to get approximate timing information of early copy number alteration events, which may be helpful in estimating the potential onset age of the disease. The confidence intervals obtained from bootstrapping can reflect the uncertainties of the estimations to some extent. We consider the incorporation of rate variation in a Bayesian framework as future work. These points have been briefly incorporated into subsection “Overview of CNETML” and Discussion of the manuscript.

- How well does the heuristic perform relative to exhaustive enumeration? The paper states that exhaustive enumeration is feasible up to 7 samples. So one can run both the heuristic and the exact version of CNETML on such data, and assess decrease in likelihood/performance due to the heuristic.

Response:
We tested both the heuristic and exhaustive tree search algorithms on simulated data with 5, 6, and 7 samples. As expected, the differences of the minimal negative log likelihood values between those obtained from the heuristic and exact version of CNETML increased with the number of samples (Supplementary Figure 4). However, the heuristic version of CNETML still had reasonably good performance. The distributions of the distances of reconstructed trees to the true trees were very similar between the heuristic and exhaustive search version, with slightly larger differences from the heuristic version on the branch score distance (Supplementary Figure 5). Moreover, the heuristic version of CNETML reconstructed the same tree topologies as the exact version on data with 5 samples and around 70% of the same tree topologies on data with 7 samples (Supplementary Table 4). These results have been incorporated into subsection “Performance on reconstructing trees and ancestral states” of the manuscript.

- How well does L-BFGS-B infer model parameters when given the right tree topology?

Response:
We tested the inference of model parameters with tree topology fixed on the simulated data with 5 samples and 1000 sites in Figure 4. The results (Supplementary Figure 10) suggest that joint estimation performed almost as well as when the tree topology was fixed. The differences of estimated and real value of the parameters (duplication/deletion rate and LUCA age) were very subtle when the mutation rate was low (0.001 per haplotype per site per year) and there was very slight underestimation under small sampling time differences when the mutation rate was high (0.01 per haplotype per site per year). These results have been briefly incorporated into subsection “Performance on jointly estimating the tree and mutation rates” of the manuscript.


- The introduction mention several methods for copy-number tree inference. However, the experimental evaluation does not include these methods (with the exception of MEDICC 2 for a restricted scenerio). Please benchmark against other methods.

Response:
We did not compare with other methods, because we emphasize the function of CNETML in jointly estimating tree topology, node ages, and mutation rates from total copy numbers and the capability of CNETML in dealing with relative copy numbers called from shallow whole genome sequencing, which is not performed by the other methods. Since we focus on building bulk sample trees, it seems not very relevant to compare with other methods designed for building mutation trees, clone trees, and single cell trees. We could compare CNETML with a basic maximum parsimony approach, but the comparisons may not add much information since the benchmark against the simulated true data has shown CNETML achieved overall good performance.
The most closely related program that can do joint estimation is PISCA (http://dx.doi.org/10.1038/s41467-017-02621-x), but it is designed for haplotype-specific copy numbers called from SNP array. Therefore, it is not a fair comparison as CNETML is designed for total copy numbers called from WGS. To check consistency, we applied CNETML on the most recent haplotype-specific copy numbers of the single crypt and whole epithelium data for two patients (391-P and 852-P) in Fig. 7 in PISCA paper, obtained from the authors. For patient 391-P, there are 52 crypt samples and 10 whole epithelium samples. For patient 852-P, there are 61 crypt samples and 11 whole epithelium samples.
Since there are subclonal WGDs in the data, we used the similar random rounding strategy as PISCA to convert absolute copy numbers into relative values. As only regions with atypical copy numbers in some samples were included in the data, we filled the missing regions in the genome with copy numbers based on the detected ploidy (rounded mean copy number) of the sample. To reduce bias, we ran random rounding 50 times and applied CNETML on each dataset. Then we computed the distances of the reconstructed trees with the trees reported in the PISCA paper. We found some inconsistences of the trees built from whole epithelium data, as the trees built on total copy numbers should be worse for patient 852-P, which was partly because those trees in the paper were generated on an older version of the copy number data (personal communication). For the single-crypt level, the reconstructed trees from both methods are very different, which is probably because of the large number of samples. Therefore, it is hard to draw a good conclusion from such comparisons.
Figure 1 The comparison of trees built by CNETML and PISCA on the single crypt and whole epithelium data from two patients 391-P and 852-P.


Minor comments:

- What are relative CNPs? Relative to the most frequent copy-number value?
Response:
Relative CNPs are copy number profiles relative to the ploidy of the genome, which is often obtained by normalised read counts relative to the median normalised read counts across the genome (https://doi.org/10.1101/2021.07.19.452658). We have clarified the definition to avoid confusions.

- Page 10, line 25. consistent => statistically consistent.
Response:
We have done the change.

- Page 10, line 42. What are unique variant sites?
Response:
We have added the definition of unique variant sites.

- Page 20, line 49. Interspersed how?
Response:
We have refined the description to be “inserted at a random position of a random chromosome”.

- Release simulated and real data to facilitate reproducibility and future benchmarking.
Response:
We have uploaded these data to Zenodo at https://doi.org/10.5281/zenodo.7313230.


[1] Zaccaria, Simone, and Benjamin J. Raphael. "Characterizing Allele- and Haplotype-Specific Copy Numbers in Single Cells with CHISEL." Nature Biotechnology, September 2, 2020.

**Second round of review**

**Reviewer 1**

The authors responded to our first comments and clearly improved the paper. However, some of their changes introduce new issues. First and foremost, now the paper references single cell genomics as a possible application. This is important, because more and more single cell data sets are getting available in cancer genomics.

The authors write “Although CNETML aims to build a bulk sample tree where each tip is a CNP from a patient bulk sample, it can be used to build trees from CNPs of subclones or single cells, since the main input is simply an integer copy number matrix where the rows can represent subclones or cells. For example, we applied CNETML on total and haplotype-specific copy numbers of subclones detected from single-cell DNA sequencing of a breast cancer patient.”

However, in the current form, there is not much evidence that CNETML can be widely applied to single cell data. For example, at some point, the authors point out the issue of running CNETML on sample sizes > 30, which contradicts its application to almost all single-cell data.

I would like to see evidence that the method is applicable to more than subclonal copy number structures derived from pseudo-bulk of single cells. If it is directly applicable to single-cell derived copy number profiles this needs to be shown with more evidence. If not, then this limitation needs to be pointed out clearly and the mentioning of single cell data should be removed in the absence of any substantial data/simulations of this kind of data.

Minor issues:

Authors write “However, even when the mean duplication/deletion size was 5 Mbp, longer than the typical size to define focal CNA (3 Mbp), the bias was not very large, and the tree topologies were still recovered well”

Please provide a source for this definition of focal CNA (seems somewhat arbitrary otherwise)

Authors write “the estimated rates and LUCA age were slightly higher with larger variances for bin-level data on larger CNAs, which is probably caused by dependencies between adjacent bins with the same copy numbers.”

I am afraid it is still quite unclear to me what you are trying to say. Can you explain what the variance refers to? Is it a consequence of CNA size, of bin-level vs segment level analysis?

It needs to be made clearer how a dependency between adjacent bins can lead to an increase in variance? Shouldn’t the observations be independent assuming they share the same copy number state? This needs to be clarified.

**Reviewer 2**

I am satisfied that the revised manuscript addresses all of my prior concerns. The authors have been quite responsive to the prior critiques, running a considerable number of additional experiments and analyses, particularly to resolve questions about the accuracy of the method and how well it generalizes to other conditions and to deviations from the model assumptions. I believe the results provide good support for the robustness and effectiveness of the approach. As before, I believe the paper is a valuable contribution to the field, making some timely and innovative contributions to methodology for an important problem in cancer genomics.

**Authors Response**

**Point-by-point responses to the reviewers’ comments:**

**Reviewer #1:** The authors responded to our first comments and clearly improved the paper. However, some of their changes introduce new issues. First and foremost, now the paper references single cell genomics as a possible application. This is important, because more and more single cell data sets are getting available in cancer genomics.

The authors write “Although CNETML aims to build a bulk sample tree where each tip is a CNP from a patient bulk sample, it can be used to build trees from CNPs of subclones or single cells, since the main input is simply an integer copy number matrix where the rows can represent subclones or cells. For example, we applied CNETML on total and haplotype-specific copy numbers of subclones detected from single-cell DNA sequencing of a breast cancer patient.”

However, in the current form, there is not much evidence that CNETML can be widely applied to single cell data. For example, at some point, the authors point out the issue of running CNETML on sample sizes > 30, which contradicts its application to almost all single-cell data.
I would like to see evidence that the method is applicable to more than subclonal copy number structures derived from pseudo-bulk of single cells. If it is directly applicable to single-cell derived copy number profiles this needs to be shown with more evidence. If not, then this limitation needs to be pointed out clearly and the mentioning of single cell data should be removed in the absence of any substantial data/simulations of this kind of data.

Response:
We acknowledge that phylogenetic inference from single cell data is becoming more and more important in cancer genomics. Given that the main input of CNETML is an integer matrix of copy numbers which is agnostic of the data generation process, it is natural that CNETML is potentially applicable to single cell data. As our method was mainly developed for bulk shallow sequencing data, we only briefly mentioned this application in Discussion but did not claim that CNETML can be widely applied to single cell data. In the first round of review, we demonstrated the application of CNETML to derived clones from single cell data (Supplementary Figure 18 and 19), following the suggestions of Reviewer 3. We are glad that this application has now been well noticed in the second round of review.
When dealing with single cell DNA sequencing data which typically include thousands of cells, scalability is a critical issue. As the motivation of our method is the availability of shallow whole genome sequencing data from multiple patient samples, we did not consider scalability as a primary design and modelling consideration. However, we did not state that CNETML had issues with sample sizes > 30. In fact, we showed that CNETML can be applied to samples of sizes up to 200 at the cost of longer running time (Supplementary Figure 5). What we wanted to emphasize in the manuscript was that CNETML is practical for bulk sequencing data, which rarely have more than 30 samples for a single patient. We apologise for this confusion and have now removed the relevant sentence in the manuscript.
In this revision, to show potential applicability of CNETML on copy number profiles of single cells rather than clones derived from single cells, we applied CNETML to two single cell datasets from breast cancer patients in paper from Minussi et al. (https://www.nature.com/articles/s41586-021-03357-x). As it is not practical to run CNETML on more than one thousand cells in the original datasets, we randomly sampled 100 cells from each dataset for illustration (Supplementary Figure 20 and 21, Supplementary Table 7). The strategy of sampling a subset of cells was used by NestedBD, a Bayesian approach to infer phylogenetic trees from copy number profiles of single cells, which randomly sampled 52 cells from a colorectal cancer patient to do the inference (https://www.biorxiv.org/content/10.1101/2022.01.16.476510v1). As seen from the alignments of tree topologies and relative total copy number heatmaps, CNETML generated three and five large groups of cells for the two patients respectively, similar to those obtained by the original paper and MEDICC2 (https://doi.org/10.1186/s13059‑022‑02794‑9). The relationships among individual cells in each group and the exact tree topologies had large bootstrap uncertainties due to the large sample size and lack of mutational information in the data.
In summary, we believe that we have shown the potential application of CNETML on single cell data. We consider improving the scalability of CNETML to thousands of cells to enhance its applicability as future work. We have incorporated these results in Discussion.

Minor issues:
Authors write “However, even when the mean duplication/deletion size was 5 Mbp, longer than the typical size to define focal CNA (3 Mbp), the bias was not very large, and the tree topologies were still recovered well”

Please provide a source for this definition of focal CNA (seems somewhat arbitrary otherwise)

Response:
We provided the source for the definition of focal CNA in the previous paragraph: “These sizes were chosen because focal CNAs are typically defined as CNAs of size no larger than 3 Mbp”, which is from the review by Krijgsman et al. (https://www.sciencedirect.com/science/article/pii/S0167488914003000). In this review, there are extensive discussions about the definition of focal CNAs, which suggests that “3 Mb” has been frequently used in literature and serves as a good operational size definition of focal CNA. To avoid confusion, we have added the reference again.

Authors write “the estimated rates and LUCA age were slightly higher with larger variances for bin-level data on larger CNAs, which is probably caused by dependencies between adjacent bins with the same copy numbers.”

I am afraid it is still quite unclear to me what you are trying to say. Can you explain what the variance refers to? Is it a consequence of CNA size, of bin-level vs segment level analysis?
It needs to be made clearer how a dependency between adjacent bins can lead to an increase in variance? Shouldn’t the observations be independent assuming they share the same copy number state? This needs to be clarified.

Response:
In the original text, the variances refer to the variances of the estimated rates and LUCA age. To highlight the differences of the estimations of mutation rates and LUCA age on bin-level and segment-level data as shown in Figure 6, we plotted the mean, standard deviation (rather than variance to scale the data for better visualization), and dispersion index (variance to mean ratio) of their values (Supplementary Figure 13).
We agree with the reviewer that the original statement was confusing. What we want to say is that the slight increase in the bias and variance of estimated rates when using bin-level data is likely due to violation of independent sites assumption as a result of larger CNAs. The changes of estimator bias and variance are known effects of pairwise site correlation (https://academic.oup.com/mbe/article/38/10/4603/6287069) and among site rate variation (https://www.sciencedirect.com/science/article/pii/0169534796100410) in phylogenetics. It is also known that large CNAs span many bins and may overlap over time, so copy numbers in adjacent bins are not independent (https://www.biorxiv.org/content/10.1101/2022.01.16.476510v1). The dependency caused by large CNAs may lead to more pairwise site correlation and higher among site rate variation in bin-level data. In contrast, segment-level data had lower dependencies among adjacent sites, since the adjacent bins with the same copy numbers were merged into a single site.
Therefore, we have altered the statement as follows: “With larger CNAs, the estimated rates obtained using bin-level data generally had slightly higher values and larger variances than those obtained using segment-level data (Figure 6B, Supplementary Figure 13). This increase of estimator bias and variance is likely due to violation of independent sites assumption which causes more pairwise site correlation [60] and higher among site rate variation [61].”

**Third round of review**

**Reviewer 1**

Lu et al have addressed the main outstanding issues to my satisfaction and this has improved the manuscript in my opinion.
The issue of single-cell applicability has been clarified, and the passage describing the bin vs segment variance is now clearer.

Only very minor thing I spotted: It would be useful to give more explicit information about what the authors mean when they say it is not practical to run CNETML on thousands of cells, whereas it is okay to run it with a subsample of 100 cells.

**Authors Response**

**Point-by-point responses to the reviewers’ comments:**

**Reviewer #1:**

We have now addressed the remaining concerns from Referee 1. In particular, we have:
expanded the text on the issue of running CNETML on thousands of cells as suggested by Referee 1, which is due to the expensive computational cost of applying full model-based phylogenetic inference to thousands of single cells that give rise to a huge number of possible tree topologies.
